# The global burden, trends, and inequalities of individuals with developmental and intellectual disabilities attributable to iodine deficiency from 1990 to 2019 and its prediction up to 2030

**DOI:** 10.3389/fnut.2024.1366525

**Published:** 2024-06-17

**Authors:** Xuesong Yang, Cheng Liu, Yanbo Liu, Zhigang He, Juan Li, Yijing Li, Yanqiong Wu, Anne Manyande, Maohui Feng, Hongbing Xiang

**Affiliations:** ^1^Hubei Key Laboratory of Geriatric Anesthesia and Perioperative Brain Health, Department of Anesthesiology and Pain Medicine, Wuhan Clinical Research Center for Geriatric Anesthesia, Tongji Hospital, Tongji Medical College, Huazhong University of Science and Technology, Wuhan, China; ^2^School of Human and Social Sciences, University of West London, London, United Kingdom; ^3^Hubei Key Laboratory of Tumor Biological Behaviors, Department of Gastrointestinal Surgery, Clinical Medical Research Center of Peritoneal Cancer of Wuhan, Clinical Cancer Study Center of Hubei Provence, Zhongnan Hospital of Wuhan University, Wuhan, China; ^4^Key Laboratory of Anesthesiology and Resuscitation (Huazhong University of Science and Technology), Ministry of Education, Wuhan, China

**Keywords:** developmental and intellectual disabilities, iodine deficiency, systematic analysis, global burden, the global burden of disease

## Abstract

**Objective:**

The objective of this study was to assess the global burden of disease for developmental and intellectual disabilities caused by iodine deficiency from 1990 to 2019.

**Methods:**

Using data from the global burden of disease (GBD) 2019, we conducted a cross-country inequity analysis to examine the worldwide burden of developmental and intellectual disabilities caused by the issue of iodine deficiency from 1990 to 2019. Absolute and relative inequality were assessed by the slope index of inequality and the concentration index, respectively. After summarising the latest evidence, we also projected the age-standardized prevalence and years lived with disability (YLD) rates up to 2030 using the BAPC and INLA packages in R statistical software.

**Results:**

In 2019, the global age-standardized prevalence and YLD rates for developmental and intellectual disabilities due to iodine deficiency were 22.54 per 100,000 population (95% UI 14.47 to 29.23) and 4.12 per 100,000 population (95% UI 2.25 to 6.4), respectively. From 1990 to 2019, the age-standardized prevalence and YLD rates of developmental and intellectual disabilities due to iodine deficiency decreased significantly. Geographic distribution showed that areas with lower socio-demographic indices (SDI) were the most affected. The correlation between higher SDI and lower prevalence highlights the role of economic and social factors in the prevalence of the disease. Cross-national inequity analysis shows that disparities persist despite improvements in health inequalities. In addition, projections suggest that the disease burden may decline until 2030.

**Conclusion:**

This research underscores the necessity for targeted interventions, such as enhancing iodine supplementation and nutritional education, especially in areas with lower SDI. We aim to provide a foundation for policymakers further to research effective preventative and potential alternative treatment strategies.

## Introduction

Iodine deficiency, a significant contributor to the global burden of disease, affects the synthesis of thyroid hormones, leading to many diseases related to metabolism and growth, that threaten people’s physical health and development (1, 2). Thyroid hormones are iodine-containing compounds, representing a combination of T_3_, T_4_, and rT_3_ (3). Among these, T_4_ is the most abundantly secreted (4), while T_3_ the most biologically active, and is approximately five times more potent than the former (5–8). The synthesis of thyroid hormones is dependent on the intake of iodine, which serves as an essential raw material for thyroid hormone production (9). Approximately 80–90% of the required iodine comes from iodide compounds found in food, primarily iodized sodium and potassium (10). The World Health Organization (WHO) recommends a daily iodine intake of 150 micrograms for adults (11). However, the physiological iodine requirements increase during pregnancy and lactation, but the daily dose should not exceed 200 micrograms (11). In addition to obtaining iodine from external sources, the iodine needed for thyroid hormone synthesis can also be recycled from iodine-containing compounds within the thyroid gland (12).

Thyroid hormones act on nearly all tissues in the body and play a crucial role in regulating various stages of promoting and maintaining growth, development, and metabolism (13), with a wide range of biological effects. During the embryonic and neonatal stages, thyroid hormones facilitate the proliferation and differentiation of neurons as well as the formation of synapses (14). Therefore, thyroid hormone deficiency during early childhood can lead to irreversible developmental disorders of the nervous system, known as cretinism (15). This condition is characterized by delayed intellectual development, stunted growth, and incomplete tooth development. Compared to the general population, individuals with intellectual disabilities are more likely to face challenges in accessing equitable healthcare and experience premature mortality (16). A study conducted in the United States by Gaylord et al. (17) reported that the difference in cost attributed to intellectual disabilities (from 2001 to 2016) would continue to yield ongoing benefits of $38 billion. Therefore, it is essential for us to comprehend the epidemiological characteristics of this disease. In humans, throughout the first three months of fetal development, the fetus is unable to synthesize thyroid hormones on its own (18). During this period, the thyroid hormones required for fetal growth and development are entirely supplied by the mother (19). Therefore, pregnant women with a history of iodine deficiency particularly need iodine supplementation to reduce the risk of cretinism (19).

Previous GBD studies have emphasized the burden of disease due to iodine deficiency or mental retardation (20–22), or have examined developmental and intellectual disabilities caused by other factors such as lead exposure (23). Rather than specifically addressing the burden of developmental and intellectual disabilities due to iodine deficiency. Despite the fact that iodine deficiency is the most prevalent and preventable cause of developmental and intellectual disabilities (2), there is a paucity of research on intellectual and developmental disabilities due to iodine deficiency based on GBD data. There is also a lack of studies predicting the burden of disease for developmental and intellectual disabilities due to iodine deficiency based on GBD data. Therefore, we used data from GBD 2019 to iodine deficiency trends in prevalence and YLD at the global, regional, and national levels, stratified by sex, age, SDI, and level of developmental and intellectual disability. Our study aims to analyze the global burden of developmental and intellectual disabilities due to iodine deficiency, provide a reference for scholars in the field, and promote the prevention of this condition.

## Materials and methods

### Definitions and data source

In the GBD study, developmental and intellectual disabilities refer to situations where an individual’s intellectual abilities are below the average. The severity of intellectual disabilities is categorized into five levels based on IQ test scores (standardized with a mean of 100), including borderline (IQ scores of 70–85), mild (IQ scores of 50–69), moderate (IQ scores of 35–49), severe (IQ scores of 20–34), and profound (IQ scores of 0–19) (24). The nonfatal iodine deficiency burden includes estimates for visible goiter (grade 2) and its associated consequences such as thyroid dysfunction, heart failure, and intellectual disability but excludes estimates for subclinical iodine deficiency or nonvisible goiter (grade 1) caused by iodine deficiency (25).

An extensive analysis was conducted to extract prevalence and YLD data related to developmental and intellectual disability associated with iodine deficiency. The analysis covered a global perspective and further profiled data from 1990 to 2019 by region, income group, and sex. Our estimates are presented in both raw values and age-standardized rates. YLD serves as a crucial metric in gauging the impact of this condition on individuals’ and societies’ quality of life. It relies on standardized disability weights assigned to each health state. The methodology for calculating YLD has been described in detail in previous studies (26–28). To compile these data, we leveraged the GBD study, which aggregates clinically informative data from various sources, including hospital records, ambulatory care (such as general practitioner visits) and health insurance claims. For each GBD cause (disease), we computed ratios of non-primary to primary diagnosis rates and ratios of outpatient to inpatient care across multiple regions. In our modeling process, we employed DisMod-MR. The strategy allowed us to generate precise estimates for each metric of interest, including prevalence and YLD, while accounting for variables such as age, sex, location, and year of analysis. We estimated the developmental and intellectual disabilities of two extended categories: severe intellectual disability and profound intellectual disability from the GBD 2019. The classification information of developmental and intellectual disabilities came from a 2008 systematic review (29). We conducted all statistical analyses and generated visualizations using R statistical software (version 4.2.3). Statistical significance was determined with a *p*-value < 0.05.

### Socio-economic status

Our estimates are categorized according to the socio-demographic index (SDI), determined by factors such as income per capita, educational attainment, and the total fertility rate among women under the age of 25 years. SDI is classified into five categories: low (< 0.46), low-middle (0.46–0.61), middle (0.61–0.69), high-middle (0.69–0.80), and high (> 0.80) (30, 31).

### Health inequalities

In this study, we utilized the concentration index (CI) and the slope index to measure the health inequalities. The slope index of inequality and concentration index, are the two established standard indicators of absolute and relative inequality, respectively (32). The slope index of inequality is determined through regression analysis of the national YLDs ratio across all age groups against a relative positional scale linked to the SDI. It’s defined as the midpoint of the population’s cumulative range ranked according to the SDI (33, 34). Heteroscedasticity is addressed using a weighted regression model. The concentration index is computed by numerically integrating the area under the Lorenz concentration curve. This curve is constructed by fitting the cumulative scores of YLDs against the cumulative relative distribution of the population according to the SDI (35). The concentration index is signed, and if the Lorenz curve is above the diagonal line, the concentration index is negative, indicating that the disease burden is concentrated in poorer countries. If the Lorenz curve is below the diagonal line, it suggests that the disease burden is concentrated in wealthier countries.

### Projections till the year 2030

We used Bayesian age-period-cohort (BAPC) models to assess and project the prevalence and YLDs rates till 2030 (36, 37). The BAPC model relies on an integrated nested Laplacian approximation to estimate marginal posterior distributions, helping circumvent some of the mixing and convergence issues associated with the traditional Bayesian method of Markov Chain Monte Carlo sampling (38). The BAPC and INLA packages in R statistical software (version 4.2.3) were used for BAPC analyses.

## Results

### The global burden of developmental and intellectual disabilities due to iodine deficiency by year and age

After controlling the effect of population and age structure, age-standardized prevalence rates for developmental and intellectual disabilities due to iodine deficiency fell by 58.54%, from 54.37 (95 % UI 38.57 to 67.63) per 100 000 population in 1990 to 22.54 (95 % UI 14.47 to 29.23) per 100 000 population in 2019 (Supplementary Table 1).

**FIGURE 1:**
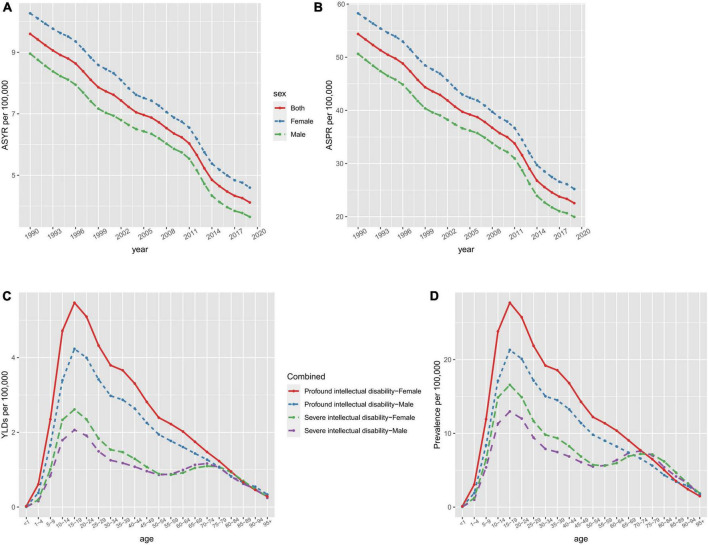
The ASYR **(A)** and ASPR **(B)** of developmental and intellectual disabilities attributable to iodine deficiency per 100,000 people from 1990 to 2019 and age-specific rates of YLDs **(C)** and prevalence **(D)** of developmental intellectual disability attributable to iodine deficiency by sex and type in 2019. ASPR, age-standardized prevalence rate; ASYR, age standardized YLDs rate.

Similarly, global age-standardized YLD rates decreased by 57.08 %, from 9.6 (95 % UI 5.61 to 14.39) per 100 000 population in 1990 to 4.12 (95 % UI 2.25 to 6.4) per 100 000 population in 2019. From 1990, the age-standardized prevalence and YLD rates showed a downward trend (Figures 1A, B). In 2019, the prevalence and YLD rates of developmental and intellectual disabilities gradually increased with age, and all reached a peak in the 15–19 age group (Figures 1C, D). Then, the prevalence and YLD rates by age declined rapidly in the 15–19 age group and slowly in those above the age of 20 to 24. For each age group, profound intellectual disability levels were higher than severe intellectual disability in 2019. Regardless of age, year, or degree of developmental and intellectual disabilities, the age-standardized prevalence and YLD rates were consistently higher in females than in males.

### Developmental and intellectual disabilities burden due to iodine deficiency based on global burden of disease regions

**FIGURE 2:**
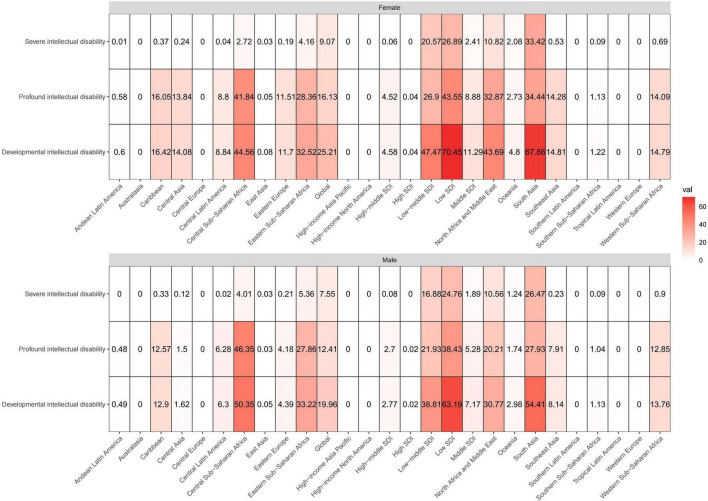
These heatmaps show the ASPR of developmental intellectual disability attributable to iodine deficiency in GBD regions by sex and severity categories in 2019. The shade of color of each block in the heatmap represents the size of the numerical value, and the figure inside represents the absolute number of the age-standardized prevalence. ASPR, age-standardized prevalence rate.

These heatmaps illustrate the distributional situation of sex and developmental and intellectual disabilities of burden due to iodine deficiency in GBD regions in 2019 (Figure 2 and Supplementary Figure 1). The shade of color of each block in the heatmap represents the size of the numerical value, and the figure inside, the absolute number of the age-standardized prevalence and YLD rates. The Low SDI region had the highest total age-standardized prevalence rates and YLD rates in both sexes, followed by South Asia and Central Sub-Saharan Africa. Profound intellectual disability accounted for the majority of the age-standardized prevalence and YLD rates of all GBD regions and the Low SDI region had the highest age-standardized prevalence rates and YLD rates for profound intellectual disability. But the lowest age-standardized prevalence rates and YLD rates were seen in South Asia.

### Geographical distribution, socio-economic disparities, and health inequalities in developmental and intellectual disabilities due to iodine deficiency

**FIGURE 3:**
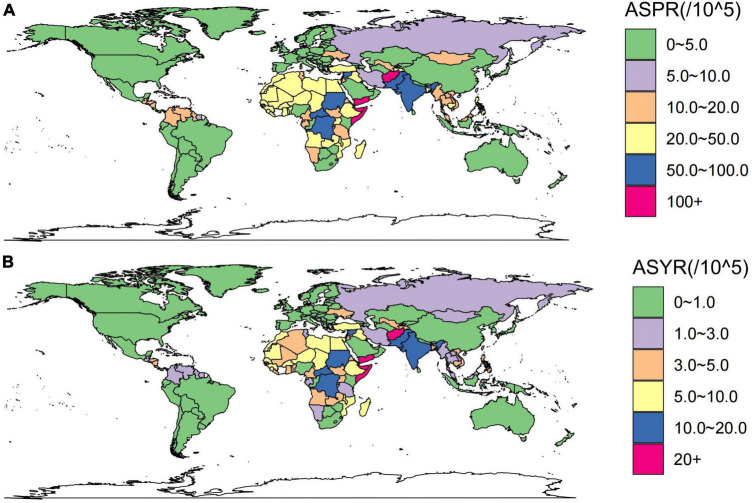
These maps show the ASPR **(A)** and ASYR **(B)** of developmental and intellectual disabilities attributable to iodine deficiency per 100,000 people in 2019. ASPR, age-standardized prevalence rate; ASYR, age standardized YLDs rate.

Figure 3 maps the distribution of the health burden of developmental and intellectual disabilities due to iodine deficiency worldwide in 2019. The age-standardized prevalence rate (Figure 3A) was highest in Somalia [162.42 (95 % UI 99.59 to 216.34) per 100 000 population], followed by Yemen [121.68 (95 % UI 65.61 to 174.69) per 100 000 population] and Afghanistan [117.09 (95 % UI 78.01 to 146.97) per 100 000 population] (Figure 3A and Supplementary Table 2). The highest age-standardized YLD rate was also found in Somalia [28.74 (95 % UI 15.49 to 45.61) per 100 000 population], followed by Yemen [21.9 (95 % UI 10.71 to 35.69) per 100 000 population] and Afghanistan [20.54 (95 % UI 12.11 to 31.52) per 100 000 population] (Figure 3B and Supplementary Table 2). HDI data in 2019 were available for 204 countries and territories, including thirty-three in the low HDI group, forty-two in low-middle SDI, forty one in middle SDI group, forty-two in high-middle SDI group, and forty-six in high HDI group.

**FIGURE 4:**
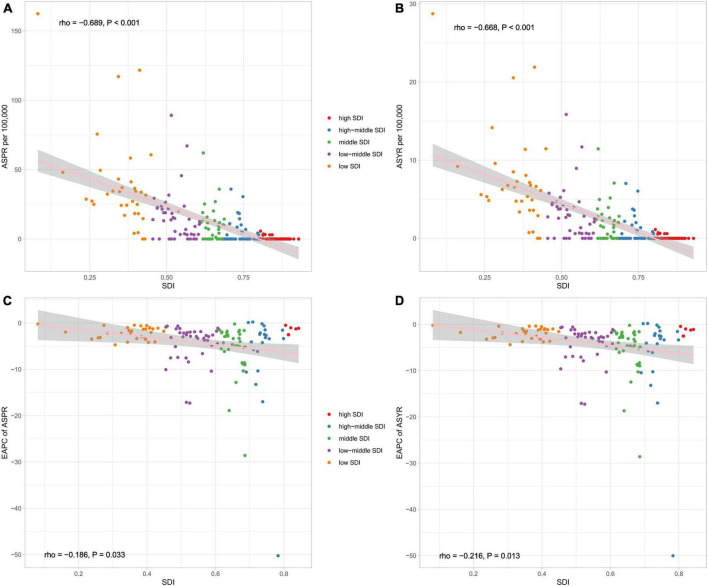
The correlation between global ASPR **(A)**, ASYR **(B)**, EAPC of ASPR **(C)**, and EAPC of ASYR **(D)** and socio-demographic index (SDI) for developmental and intellectual disabilities attributable to iodine deficiency for both sexes. ASPR, age-standardized prevalence rate; ASYR, age standardized YLDs rate. EAPC, estimated annual percentage change.

In 2019, countries with higher socio-demographic indexes tended to have lower prevalence rates than those with a low socio-demographic index (Figure 4). Spearman rank-order analysis revealed a strong, negative correlation between the age-standardized prevalence rate (rho = −0.689; *p* < 0.001) and socio-demographic index (Figure 4A), and likewise, a clear negative correlation was also seen between the age-standardized YLD rate and socio-demographic index (rho = −0.668; *p* < 0.001) (Figure 4B). The estimated annual percentage change of age-standardized prevalence and YLD rates from 1990 to 2019 showed weak correlations (rho = −0.186, *P* = 0.033; rho = −0.216, *P* = 0.013) with the socio-demographic index (Figures 4C, D).

**FIGURE 5:**
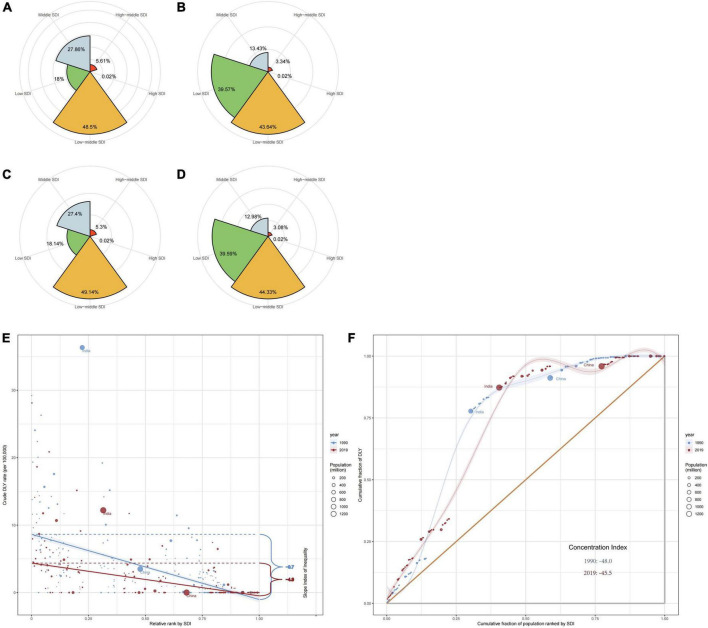
The proportion of the number of prevalence **(A)** and YLDs **(C)** in 1990 and 2019 **(B,D)** for different socio-demographic index (SDI) regions and income-related health inequality regression **(E)** and concentration curves **(F)** for YLDs of developmental and intellectual disabilities attributable to iodine deficiency across 204 counties and territories, 1990 vs. 2019.

In terms of the number of intellectual disabilities due to iodine deficiency in different SDI regions, in 1990, the low-middle SDI region had the largest number of prevalence and YLDs, accounting for 48.5 and 49.1%, followed by Middle SDI and Low SDI regions (Figures 5A, C). The high SDI region had the smallest number of prevalence and YLDs, accounting for only 0.02%. But in 2019, the proportion of the number of prevalence and YLDs cases with low SDI region increased and exceeded that of the number of cases with middle SDI region, and the other regions proportion was about the same as 1990 (Figures 5B, D).

Significant absolute and relative SDI-related inequalities in the burden of developmental and intellectual disabilities due to iodine deficiency were observed, with a disproportionately higher burden shouldered by countries with lower SDI. As illustrated by the slope index of inequality, the gap in YLDs rate between the highest and the lowest SDI country decreased from −9.7 (95% CI −10.7 to −8.7) in 1990 to −4.9 (95% CI −5.4 to −4.3) in 2019 (Figure 5E and Table 1). The results of the concentration index indicate that the between-country inequality in the distribution of the developmental and intellectual disabilities due to iodine deficiency burden declined, from −48.0 (95% CI −60.0 to −36.0) in 1990 to −45.5 (95% CI −56.8 to −34.2) in 2019 (Figure 5F and Table 1).

**TABLE 1:** Summary measures for cross-country inequalities related to SDI in YLDs of developmental and intellectual disabilities attributable to iodine deficiency.

Diseases	Health inequality metrics	Year	Value	95% CI
Developmental intellectual disabilities attributable to iodine deficiency	Slope index of inequality	1990	−9.7	−10.7 to −8.7
		2019	−4.9	−5.4 to −4.3
	Concentration index	1990	−48.0	−60.0 to −36.0
		2019	−45.5	−56.8 to −34.2

### Developmental and intellectual disabilities due to iodine deficiency projections till the year 2030

**FIGURE 6:**
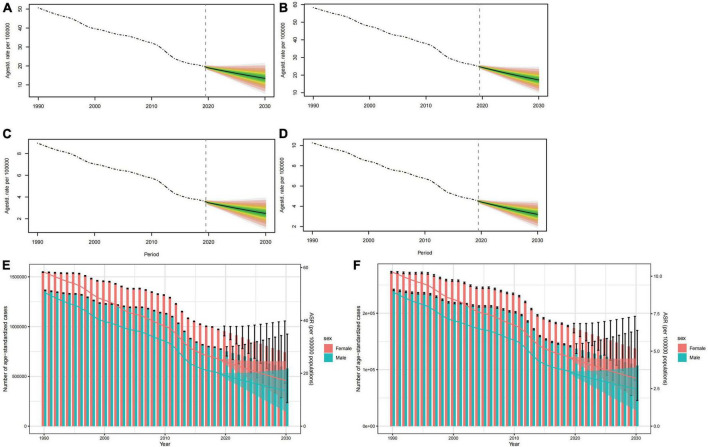
Projections of ASPR **(A,B)** and ASYR **(C,D)** in males and females from 2020 to 2030. The open dot represents the observed value, and the fan the predicted distribution between the 2.5 and 97.5% quantiles. The forecast average is shown as a solid line. The vertical dotted line indicates where the prediction begins. The projections of prevalence cases **(E)** and YLDs **(F)** by sexes of developmental and intellectual disabilities attributable to iodine deficiency from 2020 to 2030. The error bar denotes the 95% credible interval of the predictive value.

The ASPR and ASYR for both sexes are projected to see a gradual decline from 2020 to 2030, as depicted in Figures 6A–D. It is worth noting that the trend for age-specific prevalence rate for both sexes will fall across all age groups and the highest level is found in the 5–19 year age group (Supplementary Figures 2, 3). The pattern of age-specific YLDs rate closely aligns with the global age-specific prevalence rate trend (Supplementary Figures 4, 5). It is anticipated that from 2020 to 2030, both mortality cases and YLDs will diminish annually, with the numbers for females significantly outweighing those of males (Figures 6E, F).

## Discussion

This review presents a comprehensive analysis of the global burden of developmental and intellectual disabilities due to iodine deficiency from 1990 to 2019, and uncovered a promising decline in age-standardized prevalence and YLD rates over this period. It also exhibited age and sex patterns, which suggest the importance of addressing iodine deficiency during adolescence and recognizing sex-specific vulnerabilities. Geographical distribution analysis underscores the need for targeted interventions in regions with limited access to iodine-rich foods and low socio-economic status. Surprisingly, we also found correlations between SDI and lower prevalence rates. These results show that economic and social factors also affect the incidence of such a disease. Developed countries with a high level of socio-economic development have already taken effective interventions to alleviate the health burdens arising from iodine deficiency. While health inequalities show improvement, a framework for action is needed to facilitate equitable distribution.

In our study, the highest burden of developmental and intellectual disabilities due to iodine deficiency was observed in regions with low-middle SDI in 2019, and this cross-country health inequalities on iodine deficiency has been found in other studies (39). The burden is high in sub-Saharan Africa and South Asia, a finding that is consistent with that of the 2015 GBD study (40). In addition, consistent results were also obtained in a GBD-based study in 2022 (20). Between 1999 and 2000, the global prevalence, however, sharply decreased. This could likely be attributed to the proportion of the population consuming iodized salt increasing from less than 20% in 1990 to 70% in 2000 (41, 42). More importantly, the United Nations Children’s Fund (UNICEF) set a goal in 1990 to eliminate Iodine Deficiency Disorders (IDD) as a public health issue by 2000 and promoted USI worldwide (21). Although progress has been made in the elimination of iodine deficiency, over two billion people worldwide still face the risk of insufficient iodine intake (43, 44). This might be due to limited dietary diversity, poor sanitary conditions, and interactions with infectious diseases (45). We also found that the declining trend in ASPR at the global and regional levels aligns with a similar trend in ASYR.

Iodine deficiency has adverse effects on people of all age groups as the highest age-standardized prevalence rate of developmental and intellectual disabilities caused by iodine deficiency was observed in the 10–19 age group. This could be due to the increased demand for iodine during adolescence, and its decreased content derived from food and salt (46). Even a mild iodine deficiency during pregnancy can result in a lowered IQ and inferior academic performance in primary school when compared to peers (47, 48). Adolescents should ensure that they consume enough iodine every day. By consuming iodised salt or iodine-rich foods, iodine deficiency can be effectively prevented and treated, and the improvement of adolescents’ iodine nutritional status can be promoted. At the same time, adolescents should undergo regular medical check-ups, including thyroid function and blood iodine levels, in order to detect and treat iodine deficiency in a timely manner. In adults, iodine deficiency can impair cognitive functions, resulting in emotional apathy, reduced learning capacity, and decreased productivity, which in turn has adverse effects on the country’s population and economy (2). The substantial expenditures associated with providing extra resources to address intellectual disabilities place a significant burden on society, not to mention the accompanying shame and the various mental and physical illnesses and their associated complications (49). This suggests that in future research, we should conduct a thorough assessment of the costs imposed on society by intellectual disabilities. Moreover, our data also highlights that the burden of developmental and intellectual disabilities resulting from iodine deficiency is greater in females than in males, potentially because male hormones stimulate thyroid growth while female hormones have an inhibitory effect (50). Hence, the increasing trend of iodine deficiency in females is indeed a matter of concern. Females should be urged not to try to avoid weight gain by dieting, vomiting or taking laxatives, as the relative lack of food intake and excessive nutrient loss may not meet iodine requirements, leading to iodine deficiency (21). In addition, females’ iodine requirements increase by more than 50 percent during pregnancy due to the increased iodine loss from the kidneys and the developmental needs of the fetus. During breastfeeding, in order to make up for the loss of iodine in breast milk, females need more iodine intake (51). Therefore, the relevant organizations should step up health education and promotion work, so that pregnant females can correctly understand the effects of iodine deficiency diseases on their health, especially in the areas that are hardest hit by iodine deficiency diseases, and should use the community as the unit of health education and promotion, so as to raise the people’s awareness of iodine deficiency diseases.

In short, although we have made significant progress in reducing the burden of diseases caused by iodine deficiency, continued efforts are essential, especially in low-SDI regions. To further alleviate this burden, it is imperative to strengthen public health strategies, promote health education, and optimize the supply of essential nutrients.

The study serves as a vital resource for scholars and policymakers in guiding prevention efforts, with a focus on improving iodine supplementation, nutritional education, and sex-specific health initiatives in at-risk regions, while encouraging further research into effective interventions and treatments. However, our review does have some limitations. The data source of this review is generated from a GBD database, which may be subject to variations in reporting and recording across different organizations, potentially affecting data accuracy. The study also primarily focuses on the prevalence and YLD rates of developmental and intellectual disabilities due to iodine deficiency and does not delve into specific interventions and treatments. Future research should, therefore, explore effective actionable strategies for prevention and management.

## Conclusion

From 1990 to 2019, the global burden of developmental and intellectual disabilities caused by iodine deficiency has decreased, especially in regions with a high socio-demographic index (SDI). However, its burden remains high in children and adolescents, as well as in low and middle-income countries, with females experiencing a higher level than males. The findings of this study are valuable for policymakers in assessing current intervention measures and guiding future nutritional supplementation strategies to alleviate the burden of intellectual and developmental disorders caused by iodine deficiency.

## Data availability statement

The original contributions presented in this study are included in the article/Supplementary material, further inquiries can be directed to the corresponding authors.

## Ethics statement

The manuscript presents research on animals that do not require ethical approval for their study.

## Author contributions

XY: Data curation, Investigation, Software, Writing – original draft. CL: Data curation, Investigation, Software, Writing – original draft. YaL: Data curation, Investigation, Software, Writing – review & editing. ZH: Data curation, Investigation, Software, Writing – review & editing. JL: Data curation, Investigation, Writing – review & editing. YiL: Data curation, Investigation, Writing – review & editing. YW: Data curation, Formal analysis, Investigation, Writing – review & editing. AM: Conceptualization, Supervision, Writing – review & editing. MF: Conceptualization, Formal analysis, Funding acquisition, Supervision, Writing – review & editing. HX: Conceptualization, Formal analysis, Funding acquisition, Writing – original draft, Writing – review & editing.

## Supplementary material

Supplementary Figure 1 These heatmap show the ASYR of developmental intellectual disability attributable to iodine deficiency in GBD regions by sex and severity categories in 2019. The shade of color of each block in the heatmap represents the size of the numerical value, and the figure inside represents the absolute number of the age-standardized prevalence. ASYR, age standardized YLDs rate.

Supplementary Figure 2 The projections of ASPR by age for males of developmental intellectual disability attributable to iodine deficiency from 2020 to 2030. ASPR, age-standardized prevalence rate.

Supplementary Figure 3 The projections of ASPR by age for females of developmental intellectual disability attributable to iodine deficiency from 2020 to 2030. ASPR, age-standardized prevalence rate.

Supplementary Figure 4 The projections of ASYR by age for males of developmental intellectual disability attributable to iodine deficiency from 2020 to 2030. ASYR, age standardized YLDs rate.

Supplementary Figure 5 The projections of ASYR by age for females of developmental intellectual disability attributable to iodine deficiency from 2020 to 2030. ASYR, age standardized YLDs rate.
