# Supplementary material for: The global burden, trends, and inequalities of individuals with developmental and intellectual disabilities attributable to iodine deficiency from 1990 to 2019 and its prediction up to 2030
Source: Front Nutr. 2024 Jun 17;11:1366525. doi: 10.3389/fnut.2024.1366525 (PMC11215058; doi:10.3389/fnut.2024.1366525)
Supplement: Supplementary file 1 [file Table_1.DOCX]

Table S1. Prevalence and YLDs of developmental intellectual disability attributable to iodine deficiency in 1990 and 2019 for both sexes and all locations.

| location | Prevalence | | | |  | YLDs | | | |
| --- | --- | --- | --- | --- | --- | --- | --- | --- | --- |
|  | Number in 1990 | ASPR in 1990 | Number in 2019 | ASPR in 2019 |  | Number in 1990 | ASYR in 1990 | Number in 2019 | ASYR in 2019 |
| Global | 2955939 (2106946 to 3677780) | 54.37 (38.57 to 67.63) | 1751707 (1124857 to 2271089) | 22.54 (14.47 to 29.23) |  | 522410 (306451 to 782379) | 9.6 (5.61 to 14.39) | 319756 (175195 to 497334) | 4.12 (2.25 to 6.4) |
| High SDI | 583 (281 to 872) | 0.07 (0.03 to 0.1) | 345 (155 to 525) | 0.03 (0.01 to 0.05) |  | 116 (49 to 198) | 0.01 (0.01 to 0.02) | 69 (28 to 121) | 0.01 (0 to 0.01) |
| High-middle SDI | 156717 (90642 to 225218) | 13.38 (7.79 to 19.2) | 53978 (28385 to 76706) | 3.66 (1.87 to 5.23) |  | 29319 (14035 to 47695) | 2.5 (1.2 to 4.06) | 10683 (4559 to 17827) | 0.73 (0.31 to 1.22) |
| Middle SDI | 809624 (515957 to 1066429) | 47.85 (30.69 to 63.33) | 227321 (136423 to 305283) | 9.21 (5.54 to 12.33) |  | 145527 (79136 to 226511) | 8.57 (4.67 to 13.33) | 42914 (21484 to 66735) | 1.74 (0.88 to 2.71) |
| Low-middle SDI | 1452236 (1116087 to 1714400) | 131.81 (100.43 to 155.87) | 776150 (496888 to 1011954) | 43.11 (27.94 to 56.08) |  | 253294 (158121 to 370039) | 22.92 (14.17 to 33.46) | 139496 (76591 to 213017) | 7.73 (4.28 to 11.84) |
| Low SDI | 535979 (393370 to 662015) | 107.77 (77.76 to 134.49) | 693212 (449796 to 908251) | 66.82 (43.36 to 87.81) |  | 94008 (55716 to 140603) | 18.85 (11.13 to 28.33) | 126459 (69481 to 197781) | 12.1 (6.69 to 18.62) |
| Central Sub-Saharan Africa | 33952 (13714 to 58511) | 59.79 (24.55 to 102.24) | 63889 (26178 to 110615) | 47.45 (20.33 to 82.03) |  | 6304 (2225 to 11575) | 11.05 (4.15 to 20.14) | 12537 (4217 to 23321) | 9.25 (3.22 to 17.05) |
| East Asia | 221100 (108212 to 339028) | 17.65 (8.77 to 26.9) | 1113 (629 to 1836) | 0.07 (0.04 to 0.11) |  | 41334 (17883 to 69847) | 3.28 (1.47 to 5.55) | 203 (97 to 376) | 0.01 (0.01 to 0.02) |
| Eastern Europe | 18562 (10192 to 26274) | 7.92 (4.26 to 11.22) | 17841 (9696 to 25647) | 8.14 (4.29 to 11.69) |  | 3646 (1660 to 5946) | 1.56 (0.69 to 2.54) | 3517 (1537 to 5819) | 1.61 (0.69 to 2.71) |
| Eastern Sub-Saharan Africa | 136468 (84412 to 186866) | 75.55 (45.61 to 105.13) | 130571 (72058 to 188494) | 32.86 (17.56 to 48.59) |  | 24167 (12671 to 38795) | 13.38 (6.96 to 21.47) | 25147 (11435 to 41764) | 6.31 (2.78 to 10.57) |
| Andean Latin America | 367 (184 to 614) | 1.15 (0.6 to 1.83) | 345 (195 to 512) | 0.54 (0.31 to 0.81) |  | 67 (29 to 124) | 0.21 (0.1 to 0.38) | 69 (32 to 118) | 0.11 (0.05 to 0.19) |
| High-income Asia Pacific | 0 (0 to 0) | 0 (0 to 0) | 0 (0 to 0) | 0 (0 to 0) |  | 0 (0 to 0) | 0 (0 to 0) | 0 (0 to 0) | 0 (0 to 0) |
| High-income North America | 0 (0 to 0) | 0 (0 to 0) | 0 (0 to 0) | 0 (0 to 0) |  | 0 (0 to 0) | 0 (0 to 0) | 0 (0 to 0) | 0 (0 to 0) |
| Caribbean | 6448 (2299 to 10000) | 18.65 (7.08 to 28.68) | 7071 (2755 to 10372) | 14.68 (5.67 to 21.51) |  | 1261 (391 to 2172) | 3.64 (1.19 to 6.25) | 1393 (460 to 2403) | 2.89 (0.95 to 5) |
| Australasia | 0 (0 to 0) | 0 (0 to 0) | 0 (0 to 0) | 0 (0 to 0) |  | 0 (0 to 0) | 0 (0 to 0) | 0 (0 to 0) | 0 (0 to 0) |
| Central Europe | 1641 (898 to 2328) | 1.32 (0.72 to 1.87) | 0 (0 to 0) | 0 (0 to 0) |  | 323 (151 to 538) | 0.26 (0.12 to 0.43) | 0 (0 to 0) | 0 (0 to 0) |
| Central Latin America | 15976 (4197 to 25927) | 10.46 (3.2 to 16.82) | 19456 (5346 to 31795) | 7.59 (2.09 to 12.38) |  | 3172 (755 to 5697) | 2.07 (0.55 to 3.65) | 3864 (935 to 6915) | 1.51 (0.37 to 2.69) |
| Central Asia | 10568 (5261 to 17002) | 15.78 (7.99 to 25.07) | 7448 (3681 to 10907) | 7.88 (3.93 to 11.51) |  | 2031 (858 to 3613) | 3.02 (1.29 to 5.29) | 1469 (614 to 2450) | 1.55 (0.65 to 2.57) |
| North Africa and Middle East | 219123 (137683 to 298443) | 69.04 (44.33 to 93.25) | 225931 (135265 to 304999) | 36.97 (22.36 to 49.61) |  | 39759 (21393 to 62783) | 12.46 (6.78 to 19.54) | 42298 (21555 to 67264) | 6.9 (3.53 to 11.03) |
| Oceania | 467 (274 to 655) | 8.64 (5.52 to 11.75) | 435 (242 to 717) | 3.87 (2.32 to 6.03) |  | 83 (45 to 133) | 1.54 (0.88 to 2.39) | 80 (38 to 149) | 0.71 (0.37 to 1.25) |
| South Asia | 1999901 (1525749 to 2356206) | 185.37 (141.29 to 219.06) | 1136564 (746286 to 1441295) | 60.97 (40.28 to 77.26) |  | 347729 (217431 to 510003) | 32.13 (19.87 to 47.08) | 201395 (114919 to 310398) | 10.78 (6.13 to 16.48) |
| Southeast Asia | 232078 (156630 to 293450) | 54.08 (36.66 to 67.91) | 81186 (41907 to 114305) | 11.52 (5.97 to 16.24) |  | 41491 (23681 to 62921) | 9.62 (5.5 to 14.62) | 16015 (7085 to 26173) | 2.27 (1.01 to 3.69) |
| Southern Latin America | 0 (0 to 0) | 0 (0 to 0) | 0 (0 to 0) | 0 (0 to 0) |  | 0 (0 to 0) | 0 (0 to 0) | 0 (0 to 0) | 0 (0 to 0) |
| Southern Sub-Saharan Africa | 3552 (1383 to 5687) | 7.05 (2.78 to 11.42) | 944 (310 to 1545) | 1.18 (0.4 to 1.93) |  | 685 (229 to 1212) | 1.35 (0.48 to 2.4) | 185 (52 to 336) | 0.23 (0.07 to 0.42) |
| Tropical Latin America | 845 (457 to 1264) | 0.59 (0.34 to 0.87) | 0 (0 to 0) | 0 (0 to 0) |  | 156 (73 to 262) | 0.11 (0.05 to 0.18) | 0 (0 to 0) | 0 (0 to 0) |
| Western Europe | 0 (0 to 0) | 0 (0 to 0) | 0 (0 to 0) | 0 (0 to 0) |  | 0 (0 to 0) | 0 (0 to 0) | 0 (0 to 0) | 0 (0 to 0) |
| Western Sub-Saharan Africa | 54892 (27861 to 84559) | 30.71 (15.17 to 48.2) | 58912 (27622 to 89156) | 14.29 (6.59 to 22.05) |  | 10202 (4415 to 17586) | 5.71 (2.42 to 9.92) | 11582 (4807 to 19592) | 2.79 (1.11 to 4.78) |
