# Supplementary material for: The global burden, trends, and inequalities of individuals with developmental and intellectual disabilities attributable to iodine deficiency from 1990 to 2019 and its prediction up to 2030
Source: Front Nutr. 2024 Jun 17;11:1366525. doi: 10.3389/fnut.2024.1366525 (PMC11215058; doi:10.3389/fnut.2024.1366525)
Supplement: Supplementary file 2 [file Table_2.DOCX]

Table S2. Prevalence and YLDs of developmental intellectual disability attributable to iodine deficiency in 1990 and 2019 for both sexes and all locations.

| location | Prevalence | | | |  | YLDs | | | |
| --- | --- | --- | --- | --- | --- | --- | --- | --- | --- |
|  | Number in 1990 | ASPR in 1990 | Number in 2019 | ASPR in 2019 |  | Number in 1990 | ASPR in 1990 | Number in 2019 | ASPR in 2019 |
| Afghanistan | 13139 (9642 to 16194) | 128.38 (94.87 to 157.51) | 40540 (25706 to 51979) | 117.09 (78.01 to 146.97) |  | 2291 (1379 to 3471) | 22.37 (13.58 to 33.86) | 7136 (4067 to 11187) | 20.54 (12.11 to 31.52) |
| Albania | 368 (203 to 537) | 11.58 (6.57 to 16.59) | 0 (0 to 0) | 0 (0 to 0) |  | 73 (32 to 128) | 2.27 (1.08 to 3.87) | 0 (0 to 0) | 0 (0 to 0) |
| Algeria | 8797 (4365 to 14067) | 40.6 (21.07 to 62.11) | 8333 (4797 to 11944) | 20.16 (11.83 to 28.49) |  | 1627 (721 to 2893) | 7.42 (3.38 to 12.69) | 1637 (768 to 2776) | 3.94 (1.87 to 6.62) |
| American Samoa | 0 (0 to 0) | 0 (0 to 0) | 0 (0 to 0) | 0 (0 to 0) |  | 0 (0 to 0) | 0 (0 to 0) | 0 (0 to 0) | 0 (0 to 0) |
| Andorra | 0 (0 to 0) | 0 (0 to 0) | 0 (0 to 0) | 0 (0 to 0) |  | 0 (0 to 0) | 0 (0 to 0) | 0 (0 to 0) | 0 (0 to 0) |
| Angola | 4361 (1501 to 8024) | 42.26 (15.4 to 76.61) | 7103 (1979 to 12850) | 22.83 (6.75 to 41.11) |  | 831 (247 to 1635) | 7.99 (2.4 to 15.79) | 1408 (298 to 2754) | 4.51 (1.13 to 8.79) |
| Antigua and Barbuda | 6 (1 to 9) | 9.76 (2.58 to 15.47) | 5 (1 to 8) | 4.83 (0.9 to 7.85) |  | 1 (0 to 2) | 1.94 (0.41 to 3.56) | 1 (0 to 2) | 0.97 (0.16 to 1.81) |
| Argentina | 0 (0 to 0) | 0 (0 to 0) | 0 (0 to 0) | 0 (0 to 0) |  | 0 (0 to 0) | 0 (0 to 0) | 0 (0 to 0) | 0 (0 to 0) |
| Armenia | 0 (0 to 0) | 0 (0 to 0) | 0 (0 to 0) | 0 (0 to 0) |  | 0 (0 to 0) | 0 (0 to 0) | 0 (0 to 0) | 0 (0 to 0) |
| Australia | 0 (0 to 0) | 0 (0 to 0) | 0 (0 to 0) | 0 (0 to 0) |  | 0 (0 to 0) | 0 (0 to 0) | 0 (0 to 0) | 0 (0 to 0) |
| Austria | 0 (0 to 0) | 0 (0 to 0) | 0 (0 to 0) | 0 (0 to 0) |  | 0 (0 to 0) | 0 (0 to 0) | 0 (0 to 0) | 0 (0 to 0) |
| Azerbaijan | 770 (420 to 1148) | 10.9 (6.14 to 16.04) | 0 (0 to 0) | 0 (0 to 0) |  | 149 (64 to 258) | 2.1 (0.95 to 3.57) | 0 (0 to 0) | 0 (0 to 0) |
| Bahamas | 15 (2 to 26) | 5.94 (0.85 to 9.97) | 17 (4 to 29) | 4.2 (0.79 to 6.91) |  | 3 (0 to 6) | 1.19 (0.17 to 2.26) | 3 (1 to 7) | 0.84 (0.14 to 1.6) |
| Bahrain | 0 (0 to 0) | 0 (0 to 0) | 0 (0 to 0) | 0 (0 to 0) |  | 0 (0 to 0) | 0 (0 to 0) | 0 (0 to 0) | 0 (0 to 0) |
| Bangladesh | 101774 (48111 to 167015) | 99.67 (48.09 to 159.93) | 39196 (13911 to 67363) | 23.74 (8.58 to 40.71) |  | 19175 (7959 to 33417) | 18.56 (8.01 to 31.73) | 7760 (2511 to 14193) | 4.69 (1.54 to 8.58) |
| Barbados | 31 (6 to 53) | 11.96 (2.47 to 20.06) | 33 (7 to 56) | 10.12 (1.81 to 17.32) |  | 6 (1 to 12) | 2.37 (0.48 to 4.6) | 6 (1 to 13) | 2.02 (0.29 to 4) |
| Belarus | 683 (421 to 980) | 6.24 (3.72 to 9) | 466 (280 to 672) | 4.58 (2.65 to 6.83) |  | 133 (65 to 225) | 1.22 (0.58 to 2.09) | 92 (44 to 153) | 0.91 (0.43 to 1.53) |
| Belgium | 0 (0 to 0) | 0 (0 to 0) | 0 (0 to 0) | 0 (0 to 0) |  | 0 (0 to 0) | 0 (0 to 0) | 0 (0 to 0) | 0 (0 to 0) |
| Belize | 9 (5 to 13) | 5.49 (3.15 to 7.87) | 14 (7 to 20) | 3.31 (1.7 to 4.9) |  | 2 (1 to 3) | 1.09 (0.52 to 1.85) | 3 (1 to 5) | 0.66 (0.3 to 1.15) |
| Benin | 1661 (797 to 2601) | 41.28 (19.51 to 65.9) | 2589 (1343 to 3773) | 24.19 (11.67 to 35.76) |  | 307 (128 to 536) | 7.63 (3.08 to 13.54) | 513 (207 to 856) | 4.76 (1.87 to 8.05) |
| Bermuda | 3 (0 to 6) | 5.02 (0.59 to 8.66) | 2 (0 to 4) | 2.73 (0.21 to 5.01) |  | 1 (0 to 1) | 1.01 (0.11 to 1.94) | 0 (0 to 1) | 0.55 (0.04 to 1.13) |
| Bhutan | 271 (143 to 410) | 50.99 (28.79 to 74.16) | 0 (0 to 0) | 0 (0 to 0) |  | 49 (24 to 86) | 9.12 (4.67 to 15.49) | 0 (0 to 0) | 0 (0 to 0) |
| Bolivia (Plurinational State of) | 367 (184 to 614) | 7.15 (3.76 to 11.35) | 345 (195 to 512) | 3.06 (1.74 to 4.55) |  | 67 (29 to 124) | 1.3 (0.6 to 2.34) | 69 (32 to 118) | 0.61 (0.29 to 1.04) |
| Bosnia and Herzegovina | 1273 (703 to 1820) | 27.28 (15.32 to 38.82) | 0 (0 to 0) | 0 (0 to 0) |  | 250 (117 to 423) | 5.34 (2.5 to 9.02) | 0 (0 to 0) | 0 (0 to 0) |
| Botswana | 126 (60 to 186) | 12.04 (5.66 to 17.87) | 0 (0 to 0) | 0 (0 to 0) |  | 25 (10 to 42) | 2.34 (0.87 to 3.91) | 0 (0 to 0) | 0 (0 to 0) |
| Brazil | 661 (334 to 1013) | 0.47 (0.25 to 0.7) | 0 (0 to 0) | 0 (0 to 0) |  | 120 (54 to 207) | 0.09 (0.04 to 0.14) | 0 (0 to 0) | 0 (0 to 0) |
| Brunei Darussalam | 0 (0 to 0) | 0 (0 to 0) | 0 (0 to 0) | 0 (0 to 0) |  | 0 (0 to 0) | 0 (0 to 0) | 0 (0 to 0) | 0 (0 to 0) |
| Bulgaria | 0 (0 to 0) | 0 (0 to 0) | 0 (0 to 0) | 0 (0 to 0) |  | 0 (0 to 0) | 0 (0 to 0) | 0 (0 to 0) | 0 (0 to 0) |
| Burkina Faso | 5094 (2844 to 7307) | 62.44 (33.64 to 91.08) | 5148 (2691 to 7644) | 27.28 (13.56 to 41.03) |  | 919 (437 to 1504) | 11.31 (5.25 to 18.79) | 1013 (437 to 1708) | 5.33 (2.23 to 9.08) |
| Burundi | 4448 (2360 to 6575) | 84.09 (44.26 to 125.56) | 5146 (2580 to 8122) | 49.47 (24.9 to 78.78) |  | 800 (370 to 1341) | 15.11 (6.81 to 25.62) | 1008 (431 to 1744) | 9.59 (3.99 to 16.98) |
| Cabo Verde | 97 (43 to 152) | 30.31 (13.02 to 48.28) | 0 (0 to 0) | 0 (0 to 0) |  | 19 (7 to 34) | 5.98 (2.13 to 10.79) | 0 (0 to 0) | 0 (0 to 0) |
| Cambodia | 8496 (5844 to 10291) | 97.24 (66.18 to 117.73) | 3559 (2053 to 4836) | 22.19 (12.93 to 30.11) |  | 1477 (850 to 2206) | 16.86 (9.83 to 25.04) | 696 (311 to 1127) | 4.32 (1.98 to 7) |
| Cameroon | 1760 (865 to 2694) | 20.47 (9.46 to 31.42) | 4944 (2441 to 7199) | 18.95 (8.95 to 27.78) |  | 344 (142 to 594) | 3.98 (1.59 to 6.88) | 980 (394 to 1688) | 3.74 (1.52 to 6.38) |
| Canada | 0 (0 to 0) | 0 (0 to 0) | 0 (0 to 0) | 0 (0 to 0) |  | 0 (0 to 0) | 0 (0 to 0) | 0 (0 to 0) | 0 (0 to 0) |
| Central African Republic | 2265 (1041 to 3605) | 80.98 (37.62 to 130.02) | 3959 (1656 to 6705) | 75.62 (33.08 to 127.98) |  | 411 (167 to 727) | 14.66 (5.97 to 26.04) | 748 (289 to 1375) | 14.16 (5.77 to 25.38) |
| Chad | 3878 (2034 to 5707) | 69.7 (35.02 to 104.54) | 3785 (1837 to 6098) | 28.69 (13.3 to 46.71) |  | 699 (310 to 1161) | 12.6 (5.6 to 21.28) | 743 (311 to 1290) | 5.59 (2.26 to 9.79) |
| Chile | 0 (0 to 0) | 0 (0 to 0) | 0 (0 to 0) | 0 (0 to 0) |  | 0 (0 to 0) | 0 (0 to 0) | 0 (0 to 0) | 0 (0 to 0) |
| China | 221100 (108212 to 339028) | 18.27 (9.08 to 27.85) | 0 (0 to 0) | 0 (0 to 0) |  | 41334 (17883 to 69847) | 3.4 (1.52 to 5.75) | 0 (0 to 0) | 0 (0 to 0) |
| Colombia | 4510 (1140 to 7286) | 14.52 (4.26 to 23.17) | 5761 (1601 to 9714) | 11.64 (3.2 to 19.58) |  | 889 (182 to 1660) | 2.85 (0.68 to 5.21) | 1140 (248 to 2146) | 2.31 (0.52 to 4.34) |
| Comoros | 62 (37 to 92) | 17.21 (10.31 to 25.21) | 94 (55 to 136) | 13.94 (8.25 to 19.85) |  | 12 (6 to 20) | 3.25 (1.59 to 5.32) | 19 (8 to 32) | 2.75 (1.25 to 4.73) |
| Congo | 703 (228 to 1272) | 28.22 (9.54 to 50.67) | 0 (0 to 0) | 0 (0 to 0) |  | 138 (36 to 275) | 5.48 (1.5 to 10.62) | 0 (0 to 0) | 0 (0 to 0) |
| Cook Islands | 0 (0 to 0) | 0 (0 to 0) | 0 (0 to 0) | 0 (0 to 0) |  | 0 (0 to 0) | 0 (0 to 0) | 0 (0 to 0) | 0 (0 to 0) |
| Costa Rica | 418 (131 to 642) | 14.37 (4.67 to 22.1) | 0 (0 to 0) | 0 (0 to 0) |  | 84 (23 to 152) | 2.87 (0.86 to 5.16) | 0 (0 to 0) | 0 (0 to 0) |
| Côte d'Ivoire | 2456 (1087 to 3866) | 22.87 (9.76 to 36.76) | 4402 (2013 to 6547) | 18.26 (8.31 to 27.54) |  | 483 (161 to 842) | 4.46 (1.52 to 7.89) | 870 (325 to 1499) | 3.59 (1.33 to 6.16) |
| Croatia | 0 (0 to 0) | 0 (0 to 0) | 0 (0 to 0) | 0 (0 to 0) |  | 0 (0 to 0) | 0 (0 to 0) | 0 (0 to 0) | 0 (0 to 0) |
| Cuba | 1650 (487 to 2575) | 14.55 (4.45 to 22.51) | 1278 (497 to 1936) | 10.26 (3.87 to 15.78) |  | 324 (92 to 590) | 2.86 (0.83 to 5.12) | 251 (81 to 452) | 2.03 (0.65 to 3.64) |
| Cyprus | 0 (0 to 0) | 0 (0 to 0) | 0 (0 to 0) | 0 (0 to 0) |  | 0 (0 to 0) | 0 (0 to 0) | 0 (0 to 0) | 0 (0 to 0) |
| Czechia | 0 (0 to 0) | 0 (0 to 0) | 0 (0 to 0) | 0 (0 to 0) |  | 0 (0 to 0) | 0 (0 to 0) | 0 (0 to 0) | 0 (0 to 0) |
| Democratic People's Republic of Korea | 0 (0 to 0) | 0 (0 to 0) | 1113 (629 to 1836) | 3.72 (2.07 to 6.27) |  | 0 (0 to 0) | 0 (0 to 0) | 203 (97 to 376) | 0.68 (0.32 to 1.27) |
| Democratic Republic of the Congo | 26170 (10556 to 44682) | 66.1 (27.31 to 112.19) | 52439 (22085 to 90381) | 58.34 (25.36 to 101.02) |  | 4839 (1700 to 8851) | 12.17 (4.58 to 22.03) | 10304 (3573 to 19029) | 11.38 (4.07 to 20.86) |
| Denmark | 0 (0 to 0) | 0 (0 to 0) | 0 (0 to 0) | 0 (0 to 0) |  | 0 (0 to 0) | 0 (0 to 0) | 0 (0 to 0) | 0 (0 to 0) |
| Djibouti | 157 (55 to 277) | 33.39 (12.17 to 58.84) | 358 (122 to 612) | 29.34 (10.42 to 50.76) |  | 31 (9 to 60) | 6.49 (1.95 to 12.56) | 71 (20 to 137) | 5.77 (1.66 to 11.15) |
| Dominica | 12 (4 to 18) | 16.26 (5.59 to 25.04) | 6 (2 to 10) | 8.52 (2.52 to 13.48) |  | 2 (1 to 4) | 3.19 (0.95 to 5.6) | 1 (0 to 2) | 1.7 (0.41 to 3.1) |
| Dominican Republic | 1285 (408 to 2032) | 18.97 (6.34 to 30.06) | 990 (178 to 1661) | 9.02 (1.69 to 15.08) |  | 255 (62 to 457) | 3.75 (1.03 to 6.68) | 199 (26 to 374) | 1.81 (0.25 to 3.4) |
| Ecuador | 0 (0 to 0) | 0 (0 to 0) | 0 (0 to 0) | 0 (0 to 0) |  | 0 (0 to 0) | 0 (0 to 0) | 0 (0 to 0) | 0 (0 to 0) |
| Egypt | 47229 (27572 to 63893) | 90.24 (54.43 to 120.88) | 25189 (15037 to 36821) | 26.91 (16.43 to 38.64) |  | 8450 (4413 to 13459) | 16.07 (8.49 to 25.55) | 4880 (2321 to 8237) | 5.16 (2.5 to 8.51) |
| El Salvador | 1030 (441 to 1543) | 21.07 (9.24 to 31.45) | 905 (263 to 1457) | 14.28 (4.28 to 22.85) |  | 205 (74 to 361) | 4.17 (1.56 to 7.3) | 180 (44 to 342) | 2.85 (0.7 to 5.36) |
| Equatorial Guinea | 310 (123 to 542) | 70.97 (29.71 to 122.36) | 150 (11 to 316) | 9.86 (0.97 to 20.64) |  | 58 (20 to 109) | 13.07 (4.7 to 24.2) | 30 (2 to 70) | 1.95 (0.17 to 4.48) |
| Eritrea | 1295 (746 to 1787) | 53.83 (32.02 to 73.11) | 1039 (609 to 1491) | 18.34 (10.58 to 25.86) |  | 228 (118 to 368) | 9.47 (4.99 to 15.26) | 206 (95 to 344) | 3.59 (1.67 to 6.03) |
| Estonia | 65 (39 to 94) | 3.95 (2.33 to 5.86) | 42 (23 to 63) | 2.99 (1.57 to 4.47) |  | 13 (6 to 21) | 0.78 (0.37 to 1.3) | 8 (4 to 15) | 0.6 (0.26 to 1.03) |
| Eswatini | 145 (48 to 229) | 20.64 (6.93 to 34.06) | 0 (0 to 0) | 0 (0 to 0) |  | 29 (8 to 51) | 4.03 (1.2 to 7.4) | 0 (0 to 0) | 0 (0 to 0) |
| Ethiopia | 70222 (44537 to 94941) | 135 (84.04 to 186.54) | 47203 (20837 to 74597) | 43.17 (18.77 to 69.12) |  | 12368 (6594 to 19636) | 23.79 (12.51 to 37.95) | 9342 (3509 to 16169) | 8.5 (3.13 to 14.79) |
| Fiji | 23 (14 to 34) | 3.87 (2.53 to 5.43) | 0 (0 to 0) | 0 (0 to 0) |  | 5 (2 to 8) | 0.74 (0.38 to 1.24) | 0 (0 to 0) | 0 (0 to 0) |
| Finland | 0 (0 to 0) | 0 (0 to 0) | 0 (0 to 0) | 0 (0 to 0) |  | 0 (0 to 0) | 0 (0 to 0) | 0 (0 to 0) | 0 (0 to 0) |
| France | 0 (0 to 0) | 0 (0 to 0) | 0 (0 to 0) | 0 (0 to 0) |  | 0 (0 to 0) | 0 (0 to 0) | 0 (0 to 0) | 0 (0 to 0) |
| Gabon | 143 (22 to 275) | 14.25 (2.56 to 27.39) | 239 (41 to 449) | 13.23 (2.38 to 24.65) |  | 28 (4 to 59) | 2.8 (0.47 to 5.79) | 47 (7 to 97) | 2.62 (0.42 to 5.37) |
| Gambia | 350 (151 to 575) | 38.83 (16.65 to 65.45) | 756 (324 to 1199) | 34.48 (14.31 to 55.8) |  | 69 (24 to 123) | 7.56 (2.71 to 13.73) | 150 (52 to 265) | 6.8 (2.32 to 12.16) |
| Georgia | 0 (0 to 0) | 0 (0 to 0) | 0 (0 to 0) | 0 (0 to 0) |  | 0 (0 to 0) | 0 (0 to 0) | 0 (0 to 0) | 0 (0 to 0) |
| Germany | 0 (0 to 0) | 0 (0 to 0) | 0 (0 to 0) | 0 (0 to 0) |  | 0 (0 to 0) | 0 (0 to 0) | 0 (0 to 0) | 0 (0 to 0) |
| Ghana | 5662 (2437 to 9548) | 40.39 (17.26 to 69.17) | 7508 (2810 to 12274) | 23.67 (8.77 to 39.21) |  | 1106 (406 to 2034) | 7.81 (2.84 to 14.46) | 1488 (476 to 2710) | 4.68 (1.53 to 8.58) |
| Greece | 0 (0 to 0) | 0 (0 to 0) | 0 (0 to 0) | 0 (0 to 0) |  | 0 (0 to 0) | 0 (0 to 0) | 0 (0 to 0) | 0 (0 to 0) |
| Greenland | 0 (0 to 0) | 0 (0 to 0) | 0 (0 to 0) | 0 (0 to 0) |  | 0 (0 to 0) | 0 (0 to 0) | 0 (0 to 0) | 0 (0 to 0) |
| Grenada | 14 (5 to 21) | 17.17 (5.99 to 26.32) | 8 (2 to 12) | 6.87 (1.8 to 10.74) |  | 3 (1 to 5) | 3.36 (1.01 to 6.06) | 2 (0 to 3) | 1.37 (0.33 to 2.59) |
| Guam | 0 (0 to 0) | 0 (0 to 0) | 0 (0 to 0) | 0 (0 to 0) |  | 0 (0 to 0) | 0 (0 to 0) | 0 (0 to 0) | 0 (0 to 0) |
| Guatemala | 1193 (474 to 1806) | 17.44 (7.84 to 25.96) | 2610 (773 to 4097) | 14.82 (4.71 to 23.23) |  | 236 (80 to 411) | 3.42 (1.28 to 5.87) | 518 (131 to 927) | 2.93 (0.79 to 5.18) |
| Guinea | 3924 (1695 to 6431) | 63.23 (27.29 to 104.43) | 4002 (1779 to 6316) | 34.55 (15 to 55.4) |  | 719 (279 to 1273) | 11.61 (4.58 to 20.95) | 791 (297 to 1420) | 6.78 (2.49 to 12.23) |
| Guinea-Bissau | 598 (260 to 969) | 61.3 (26.94 to 99.5) | 678 (300 to 1055) | 37.05 (16.13 to 58.39) |  | 111 (42 to 196) | 11.29 (4.33 to 20.05) | 134 (47 to 235) | 7.28 (2.62 to 12.79) |
| Guyana | 191 (81 to 287) | 26 (11.49 to 39.58) | 110 (38 to 168) | 13.97 (5.04 to 21.56) |  | 38 (14 to 65) | 5.09 (1.88 to 8.73) | 22 (6 to 38) | 2.76 (0.79 to 4.81) |
| Haiti | 2131 (972 to 3313) | 38.06 (17.56 to 59.4) | 3750 (1714 to 5449) | 31.59 (14.57 to 45.74) |  | 409 (152 to 708) | 7.21 (2.71 to 12.36) | 734 (279 to 1279) | 6.12 (2.34 to 10.5) |
| Honduras | 950 (394 to 1419) | 23.09 (10.25 to 34.56) | 1846 (621 to 2807) | 18.95 (6.8 to 28.75) |  | 188 (67 to 322) | 4.55 (1.69 to 7.8) | 368 (96 to 650) | 3.77 (1.11 to 6.55) |
| Hungary | 0 (0 to 0) | 0 (0 to 0) | 0 (0 to 0) | 0 (0 to 0) |  | 0 (0 to 0) | 0 (0 to 0) | 0 (0 to 0) | 0 (0 to 0) |
| Iceland | 0 (0 to 0) | 0 (0 to 0) | 0 (0 to 0) | 0 (0 to 0) |  | 0 (0 to 0) | 0 (0 to 0) | 0 (0 to 0) | 0 (0 to 0) |
| India | 1798501 (1407581 to 2102811) | 211.29 (164.11 to 247.28) | 971905 (651141 to 1203304) | 67.09 (44.95 to 83.06) |  | 310817 (199727 to 450976) | 36.41 (23 to 52.91) | 169696 (99099 to 253807) | 11.69 (6.81 to 17.49) |
| Indonesia | 81821 (59726 to 101467) | 48.34 (35.62 to 59.04) | 3254 (1938 to 4786) | 1.2 (0.72 to 1.76) |  | 14258 (8452 to 21247) | 8.39 (5.11 to 12.5) | 618 (289 to 1027) | 0.23 (0.11 to 0.38) |
| Iran (Islamic Republic of) | 17215 (12484 to 22359) | 33.47 (25.29 to 42.39) | 7636 (4631 to 10853) | 8.89 (5.46 to 12.54) |  | 3021 (1769 to 4547) | 5.85 (3.51 to 8.76) | 1499 (750 to 2509) | 1.74 (0.87 to 2.88) |
| Iraq | 7022 (3702 to 10529) | 47.22 (25.47 to 71.51) | 14654 (7601 to 21171) | 35.87 (19 to 51.12) |  | 1365 (590 to 2327) | 9.05 (4.04 to 15.29) | 2912 (1302 to 4924) | 7.07 (3.21 to 11.79) |
| Ireland | 0 (0 to 0) | 0 (0 to 0) | 0 (0 to 0) | 0 (0 to 0) |  | 0 (0 to 0) | 0 (0 to 0) | 0 (0 to 0) | 0 (0 to 0) |
| Israel | 0 (0 to 0) | 0 (0 to 0) | 0 (0 to 0) | 0 (0 to 0) |  | 0 (0 to 0) | 0 (0 to 0) | 0 (0 to 0) | 0 (0 to 0) |
| Italy | 0 (0 to 0) | 0 (0 to 0) | 0 (0 to 0) | 0 (0 to 0) |  | 0 (0 to 0) | 0 (0 to 0) | 0 (0 to 0) | 0 (0 to 0) |
| Jamaica | 367 (130 to 559) | 16.23 (5.71 to 24.95) | 308 (104 to 471) | 10.28 (3.52 to 15.81) |  | 73 (22 to 130) | 3.21 (0.98 to 5.69) | 61 (17 to 112) | 2.04 (0.57 to 3.65) |
| Japan | 0 (0 to 0) | 0 (0 to 0) | 0 (0 to 0) | 0 (0 to 0) |  | 0 (0 to 0) | 0 (0 to 0) | 0 (0 to 0) | 0 (0 to 0) |
| Jordan | 924 (549 to 1443) | 29.9 (18.43 to 43.61) | 2134 (1293 to 3045) | 19.06 (11.93 to 26.87) |  | 180 (84 to 311) | 5.71 (2.78 to 9.6) | 422 (197 to 716) | 3.74 (1.78 to 6.21) |
| Kazakhstan | 2473 (1110 to 3846) | 15.42 (7 to 24.01) | 0 (0 to 0) | 0 (0 to 0) |  | 483 (186 to 858) | 3 (1.18 to 5.32) | 0 (0 to 0) | 0 (0 to 0) |
| Kenya | 0 (0 to 0) | 0 (0 to 0) | 0 (0 to 0) | 0 (0 to 0) |  | 0 (0 to 0) | 0 (0 to 0) | 0 (0 to 0) | 0 (0 to 0) |
| Kiribati | 7 (4 to 10) | 11.01 (7.25 to 14.94) | 6 (3 to 9) | 5.46 (3.29 to 8.19) |  | 1 (1 to 2) | 1.94 (1.06 to 3.09) | 1 (0 to 2) | 0.98 (0.51 to 1.67) |
| Kuwait | 0 (0 to 0) | 0 (0 to 0) | 0 (0 to 0) | 0 (0 to 0) |  | 0 (0 to 0) | 0 (0 to 0) | 0 (0 to 0) | 0 (0 to 0) |
| Kyrgyzstan | 0 (0 to 0) | 0 (0 to 0) | 0 (0 to 0) | 0 (0 to 0) |  | 0 (0 to 0) | 0 (0 to 0) | 0 (0 to 0) | 0 (0 to 0) |
| Lao People's Democratic Republic | 2050 (1614 to 2500) | 60.16 (47.85 to 72.3) | 890 (538 to 1276) | 13.15 (7.99 to 18.63) |  | 355 (215 to 532) | 10.39 (6.35 to 15.61) | 176 (78 to 301) | 2.57 (1.18 to 4.32) |
| Latvia | 108 (63 to 155) | 3.83 (2.17 to 5.6) | 67 (37 to 97) | 3.21 (1.71 to 4.79) |  | 21 (10 to 36) | 0.76 (0.36 to 1.28) | 13 (6 to 22) | 0.64 (0.28 to 1.09) |
| Lebanon | 0 (0 to 0) | 0 (0 to 0) | 0 (0 to 0) | 0 (0 to 0) |  | 0 (0 to 0) | 0 (0 to 0) | 0 (0 to 0) | 0 (0 to 0) |
| Lesotho | 1115 (449 to 1903) | 62.97 (27.04 to 105.39) | 664 (244 to 1106) | 31.63 (12 to 53.04) |  | 212 (74 to 395) | 11.78 (4.46 to 21.62) | 130 (41 to 248) | 6.11 (1.9 to 11.72) |
| Liberia | 687 (377 to 972) | 41.17 (22.21 to 58.99) | 1008 (578 to 1444) | 24.36 (14.06 to 34.86) |  | 123 (61 to 201) | 7.36 (3.6 to 12.17) | 199 (92 to 333) | 4.76 (2.25 to 7.81) |
| Libya | 1027 (517 to 1490) | 27.79 (14.65 to 39.56) | 2536 (1306 to 3656) | 35.93 (19.18 to 51.51) |  | 203 (85 to 347) | 5.45 (2.32 to 9.12) | 499 (222 to 834) | 7.03 (3.19 to 11.59) |
| Lithuania | 145 (85 to 211) | 3.76 (2.2 to 5.52) | 92 (51 to 133) | 3 (1.59 to 4.5) |  | 29 (14 to 48) | 0.74 (0.36 to 1.24) | 18 (8 to 30) | 0.6 (0.26 to 1.01) |
| Luxembourg | 0 (0 to 0) | 0 (0 to 0) | 0 (0 to 0) | 0 (0 to 0) |  | 0 (0 to 0) | 0 (0 to 0) | 0 (0 to 0) | 0 (0 to 0) |
| Madagascar | 5017 (2353 to 7920) | 49.48 (23.45 to 77.77) | 9014 (4189 to 13737) | 36.43 (16.62 to 56.33) |  | 920 (377 to 1619) | 9.03 (3.78 to 15.95) | 1783 (717 to 3025) | 7.15 (2.82 to 12.33) |
| Malawi | 7214 (4143 to 10353) | 81.99 (45.72 to 119.32) | 7155 (3503 to 10746) | 41.18 (19.11 to 63.52) |  | 1286 (642 to 2162) | 14.6 (7.22 to 23.97) | 1411 (568 to 2421) | 8.06 (3.19 to 14.01) |
| Malaysia | 5704 (2560 to 8701) | 33.29 (14.95 to 51.3) | 4843 (1685 to 7810) | 14.53 (5.21 to 23.41) |  | 1127 (417 to 1982) | 6.53 (2.43 to 11.51) | 963 (282 to 1744) | 2.88 (0.87 to 5.15) |
| Maldives | 49 (27 to 73) | 28.3 (16.05 to 41.77) | 39 (21 to 56) | 6.85 (3.82 to 9.83) |  | 9 (4 to 16) | 5.41 (2.53 to 9.14) | 8 (3 to 13) | 1.36 (0.63 to 2.26) |
| Mali | 4137 (2359 to 5904) | 56.07 (31.07 to 79.99) | 4394 (2379 to 6628) | 24.94 (13.03 to 37.69) |  | 744 (363 to 1207) | 10.11 (4.79 to 16.49) | 861 (387 to 1445) | 4.85 (2.14 to 8.26) |
| Malta | 0 (0 to 0) | 0 (0 to 0) | 0 (0 to 0) | 0 (0 to 0) |  | 0 (0 to 0) | 0 (0 to 0) | 0 (0 to 0) | 0 (0 to 0) |
| Marshall Islands | 2 (1 to 4) | 6.82 (4.26 to 10.49) | 2 (1 to 2) | 3.22 (2.12 to 4.7) |  | 0 (0 to 1) | 1.25 (0.65 to 2.17) | 0 (0 to 1) | 0.6 (0.32 to 1) |
| Mauritania | 688 (280 to 1156) | 35.3 (14.36 to 60.39) | 1059 (397 to 1742) | 26.5 (9.85 to 44.62) |  | 135 (47 to 245) | 6.89 (2.4 to 12.68) | 210 (67 to 379) | 5.25 (1.66 to 9.58) |
| Mauritius | 258 (139 to 361) | 23.76 (13.25 to 33.45) | 161 (77 to 233) | 11.04 (5.35 to 16.05) |  | 51 (23 to 83) | 4.64 (2.09 to 7.58) | 32 (12 to 55) | 2.18 (0.85 to 3.79) |
| Mexico | 4827 (783 to 8438) | 6.09 (1.32 to 10.43) | 3482 (728 to 6020) | 2.72 (0.57 to 4.7) |  | 961 (144 to 1861) | 1.21 (0.23 to 2.27) | 692 (125 to 1312) | 0.54 (0.1 to 1.02) |
| Micronesia (Federated States of) | 5 (3 to 9) | 7.36 (4.66 to 11.06) | 3 (2 to 5) | 3.49 (2.28 to 5) |  | 1 (0 to 2) | 1.34 (0.7 to 2.27) | 1 (0 to 1) | 0.65 (0.35 to 1.06) |
| Monaco | 0 (0 to 0) | 0 (0 to 0) | 0 (0 to 0) | 0 (0 to 0) |  | 0 (0 to 0) | 0 (0 to 0) | 0 (0 to 0) | 0 (0 to 0) |
| Mongolia | 580 (274 to 950) | 29.29 (14.36 to 46.59) | 351 (176 to 513) | 10.58 (5.41 to 15.34) |  | 109 (44 to 189) | 5.46 (2.4 to 9.34) | 70 (29 to 121) | 2.09 (0.86 to 3.54) |
| Montenegro | 0 (0 to 0) | 0 (0 to 0) | 0 (0 to 0) | 0 (0 to 0) |  | 0 (0 to 0) | 0 (0 to 0) | 0 (0 to 0) | 0 (0 to 0) |
| Morocco | 26497 (13199 to 40359) | 109.11 (56.04 to 163.94) | 16660 (8791 to 23671) | 45.63 (24.39 to 64.69) |  | 4861 (2161 to 8260) | 19.9 (8.89 to 33.77) | 3282 (1368 to 5392) | 8.95 (3.79 to 14.61) |
| Mozambique | 14327 (9426 to 18934) | 117.48 (74.84 to 159.12) | 7552 (4135 to 11031) | 32.33 (17.1 to 48.87) |  | 2529 (1377 to 3946) | 20.74 (11.08 to 32.85) | 1482 (656 to 2509) | 6.25 (2.8 to 10.67) |
| Myanmar | 31211 (25632 to 36098) | 84.96 (69.03 to 97.02) | 8480 (4861 to 11917) | 15.41 (8.95 to 21.7) |  | 5390 (3334 to 7672) | 14.62 (9.13 to 20.83) | 1668 (775 to 2847) | 3.02 (1.41 to 5.11) |
| Namibia | 226 (77 to 351) | 17.5 (5.92 to 28) | 280 (66 to 444) | 11.96 (2.95 to 19.23) |  | 44 (12 to 80) | 3.42 (0.98 to 6.23) | 56 (11 to 102) | 2.38 (0.51 to 4.34) |
| Nauru | 0 (0 to 0) | 0 (0 to 0) | 0 (0 to 0) | 0 (0 to 0) |  | 0 (0 to 0) | 0 (0 to 0) | 0 (0 to 0) | 0 (0 to 0) |
| Nepal | 0 (0 to 0) | 0 (0 to 0) | 0 (0 to 0) | 0 (0 to 0) |  | 0 (0 to 0) | 0 (0 to 0) | 0 (0 to 0) | 0 (0 to 0) |
| Netherlands | 0 (0 to 0) | 0 (0 to 0) | 0 (0 to 0) | 0 (0 to 0) |  | 0 (0 to 0) | 0 (0 to 0) | 0 (0 to 0) | 0 (0 to 0) |
| New Zealand | 0 (0 to 0) | 0 (0 to 0) | 0 (0 to 0) | 0 (0 to 0) |  | 0 (0 to 0) | 0 (0 to 0) | 0 (0 to 0) | 0 (0 to 0) |
| Nicaragua | 715 (282 to 1059) | 20.83 (8.96 to 30.7) | 1210 (411 to 1871) | 18.4 (6.47 to 28.45) |  | 142 (51 to 250) | 4.12 (1.54 to 7.01) | 241 (71 to 426) | 3.66 (1.1 to 6.49) |
| Niger | 6164 (3239 to 9032) | 79.35 (40.73 to 118.97) | 9600 (4561 to 16437) | 47.94 (22 to 81.82) |  | 1106 (517 to 1824) | 14.3 (6.45 to 24.06) | 1854 (764 to 3375) | 9.21 (3.58 to 16.93) |
| Nigeria | 11915 (5781 to 18634) | 14.06 (6.57 to 22.5) | 0 (0 to 0) | 0 (0 to 0) |  | 2218 (917 to 3893) | 2.62 (1.07 to 4.63) | 0 (0 to 0) | 0 (0 to 0) |
| Niue | 0 (0 to 0) | 3.21 (2.05 to 4.47) | 0 (0 to 0) | 0 (0 to 0) |  | 0 (0 to 0) | 0.62 (0.32 to 1.03) | 0 (0 to 0) | 0 (0 to 0) |
| North Macedonia | 0 (0 to 0) | 0 (0 to 0) | 0 (0 to 0) | 0 (0 to 0) |  | 0 (0 to 0) | 0 (0 to 0) | 0 (0 to 0) | 0 (0 to 0) |
| Northern Mariana Islands | 0 (0 to 0) | 0 (0 to 0) | 0 (0 to 0) | 0 (0 to 0) |  | 0 (0 to 0) | 0 (0 to 0) | 0 (0 to 0) | 0 (0 to 0) |
| Norway | 0 (0 to 0) | 0 (0 to 0) | 0 (0 to 0) | 0 (0 to 0) |  | 0 (0 to 0) | 0 (0 to 0) | 0 (0 to 0) | 0 (0 to 0) |
| Oman | 302 (171 to 431) | 19.04 (11.34 to 26.57) | 0 (0 to 0) | 0 (0 to 0) |  | 59 (26 to 103) | 3.65 (1.72 to 6.13) | 0 (0 to 0) | 0 (0 to 0) |
| Pakistan | 99357 (63100 to 134564) | 97.94 (61.95 to 133.01) | 125463 (66763 to 188853) | 60.69 (32.97 to 90.92) |  | 17689 (9630 to 27839) | 17.37 (9.41 to 27.66) | 23938 (11478 to 40742) | 11.45 (5.54 to 19.45) |
| Palau | 0 (0 to 1) | 2.98 (1.86 to 4.22) | 0 (0 to 0) | 0 (0 to 0) |  | 0 (0 to 0) | 0.58 (0.3 to 0.97) | 0 (0 to 0) | 0 (0 to 0) |
| Palestine | 881 (568 to 1151) | 51.3 (35.46 to 65.79) | 0 (0 to 0) | 0 (0 to 0) |  | 153 (87 to 240) | 8.89 (5.24 to 13.72) | 0 (0 to 0) | 0 (0 to 0) |
| Panama | 262 (88 to 399) | 11.18 (4.14 to 16.77) | 0 (0 to 0) | 0 (0 to 0) |  | 52 (14 to 92) | 2.23 (0.69 to 3.9) | 0 (0 to 0) | 0 (0 to 0) |
| Papua New Guinea | 347 (191 to 492) | 10.13 (6.23 to 13.93) | 326 (171 to 564) | 4.07 (2.34 to 6.5) |  | 61 (31 to 98) | 1.78 (0.98 to 2.79) | 60 (28 to 116) | 0.74 (0.38 to 1.33) |
| Paraguay | 183 (96 to 272) | 5.23 (2.93 to 7.59) | 0 (0 to 0) | 0 (0 to 0) |  | 37 (16 to 62) | 1.05 (0.47 to 1.78) | 0 (0 to 0) | 0 (0 to 0) |
| Peru | 0 (0 to 0) | 0 (0 to 0) | 0 (0 to 0) | 0 (0 to 0) |  | 0 (0 to 0) | 0 (0 to 0) | 0 (0 to 0) | 0 (0 to 0) |
| Philippines | 33047 (19075 to 47497) | 56.71 (32.81 to 80.79) | 27767 (12762 to 40190) | 25.21 (11.87 to 36.52) |  | 6037 (2932 to 9988) | 10.32 (5.04 to 16.96) | 5490 (2168 to 9168) | 4.97 (1.96 to 8.25) |
| Poland | 0 (0 to 0) | 0 (0 to 0) | 0 (0 to 0) | 0 (0 to 0) |  | 0 (0 to 0) | 0 (0 to 0) | 0 (0 to 0) | 0 (0 to 0) |
| Portugal | 0 (0 to 0) | 0 (0 to 0) | 0 (0 to 0) | 0 (0 to 0) |  | 0 (0 to 0) | 0 (0 to 0) | 0 (0 to 0) | 0 (0 to 0) |
| Puerto Rico | 262 (52 to 429) | 7.16 (1.44 to 11.74) | 142 (26 to 240) | 3.58 (0.48 to 6.12) |  | 52 (8 to 98) | 1.43 (0.24 to 2.68) | 29 (4 to 55) | 0.72 (0.08 to 1.4) |
| Qatar | 0 (0 to 0) | 0 (0 to 0) | 0 (0 to 0) | 0 (0 to 0) |  | 0 (0 to 0) | 0 (0 to 0) | 0 (0 to 0) | 0 (0 to 0) |
| Republic of Korea | 0 (0 to 0) | 0 (0 to 0) | 0 (0 to 0) | 0 (0 to 0) |  | 0 (0 to 0) | 0 (0 to 0) | 0 (0 to 0) | 0 (0 to 0) |
| Republic of Moldova | 115 (68 to 168) | 2.59 (1.54 to 3.78) | 118 (69 to 173) | 2.95 (1.72 to 4.37) |  | 23 (11 to 37) | 0.51 (0.25 to 0.83) | 24 (11 to 40) | 0.59 (0.28 to 0.99) |
| Romania | 0 (0 to 0) | 0 (0 to 0) | 0 (0 to 0) | 0 (0 to 0) |  | 0 (0 to 0) | 0 (0 to 0) | 0 (0 to 0) | 0 (0 to 0) |
| Russian Federation | 8802 (4855 to 12586) | 5.61 (3.09 to 8.15) | 8696 (4544 to 12709) | 5.65 (2.9 to 8.35) |  | 1731 (818 to 2858) | 1.1 (0.52 to 1.83) | 1725 (781 to 2890) | 1.12 (0.48 to 1.9) |
| Rwanda | 0 (0 to 0) | 0 (0 to 0) | 0 (0 to 0) | 0 (0 to 0) |  | 0 (0 to 0) | 0 (0 to 0) | 0 (0 to 0) | 0 (0 to 0) |
| Saint Kitts and Nevis | 5 (1 to 7) | 11.74 (3.43 to 18.12) | 3 (1 to 5) | 4.74 (0.83 to 7.82) |  | 1 (0 to 2) | 2.31 (0.55 to 4.14) | 1 (0 to 1) | 0.95 (0.17 to 1.79) |
| Saint Lucia | 8 (4 to 12) | 6.57 (3.44 to 9.5) | 8 (4 to 12) | 4.12 (1.91 to 6.13) |  | 2 (1 to 3) | 1.3 (0.56 to 2.17) | 2 (1 to 3) | 0.83 (0.31 to 1.44) |
| Saint Vincent and the Grenadines | 18 (7 to 28) | 17.8 (6.47 to 27.27) | 10 (3 to 15) | 8.01 (2.6 to 12.36) |  | 4 (1 to 6) | 3.48 (1.12 to 6.15) | 2 (1 to 4) | 1.6 (0.46 to 2.95) |
| Samoa | 0 (0 to 0) | 0 (0 to 0) | 0 (0 to 0) | 0 (0 to 0) |  | 0 (0 to 0) | 0 (0 to 0) | 0 (0 to 0) | 0 (0 to 0) |
| San Marino | 0 (0 to 0) | 0 (0 to 0) | 0 (0 to 0) | 0 (0 to 0) |  | 0 (0 to 0) | 0 (0 to 0) | 0 (0 to 0) | 0 (0 to 0) |
| Sao Tome and Principe | 25 (13 to 38) | 25.19 (11.82 to 38.06) | 39 (18 to 57) | 19.61 (8.83 to 29.07) |  | 5 (2 to 9) | 4.95 (2.05 to 8.39) | 8 (3 to 13) | 3.87 (1.38 to 6.64) |
| Saudi Arabia | 0 (0 to 0) | 0 (0 to 0) | 0 (0 to 0) | 0 (0 to 0) |  | 0 (0 to 0) | 0 (0 to 0) | 0 (0 to 0) | 0 (0 to 0) |
| Senegal | 2525 (1117 to 4229) | 37.09 (15.65 to 63.3) | 3898 (1627 to 6156) | 27.07 (11.15 to 43.39) |  | 488 (175 to 917) | 7.12 (2.57 to 13.25) | 772 (265 to 1350) | 5.34 (1.73 to 9.41) |
| Serbia | 0 (0 to 0) | 0 (0 to 0) | 0 (0 to 0) | 0 (0 to 0) |  | 0 (0 to 0) | 0 (0 to 0) | 0 (0 to 0) | 0 (0 to 0) |
| Seychelles | 13 (6 to 18) | 18.06 (9.53 to 26.12) | 0 (0 to 0) | 0 (0 to 0) |  | 2 (1 to 4) | 3.54 (1.57 to 5.94) | 0 (0 to 0) | 0 (0 to 0) |
| Sierra Leone | 1698 (854 to 2511) | 51.27 (24.55 to 77.89) | 2567 (1255 to 4223) | 34.19 (16.56 to 56.32) |  | 308 (140 to 527) | 9.32 (4.15 to 16.22) | 496 (200 to 868) | 6.57 (2.59 to 11.7) |
| Singapore | 0 (0 to 0) | 0 (0 to 0) | 0 (0 to 0) | 0 (0 to 0) |  | 0 (0 to 0) | 0 (0 to 0) | 0 (0 to 0) | 0 (0 to 0) |
| Slovakia | 0 (0 to 0) | 0 (0 to 0) | 0 (0 to 0) | 0 (0 to 0) |  | 0 (0 to 0) | 0 (0 to 0) | 0 (0 to 0) | 0 (0 to 0) |
| Slovenia | 0 (0 to 0) | 0 (0 to 0) | 0 (0 to 0) | 0 (0 to 0) |  | 0 (0 to 0) | 0 (0 to 0) | 0 (0 to 0) | 0 (0 to 0) |
| Solomon Islands | 17 (10 to 25) | 6.6 (4.4 to 9.16) | 24 (14 to 36) | 4.56 (2.9 to 6.56) |  | 3 (2 to 5) | 1.17 (0.66 to 1.83) | 4 (2 to 7) | 0.81 (0.45 to 1.34) |
| Somalia | 11976 (8010 to 15759) | 169.47 (111.72 to 225.98) | 32276 (19999 to 43027) | 162.42 (99.59 to 216.34) |  | 2087 (1146 to 3274) | 29.52 (16.35 to 46.59) | 5721 (3071 to 9097) | 28.74 (15.49 to 45.61) |
| South Africa | 1940 (728 to 3074) | 5.4 (2.03 to 8.67) | 0 (0 to 0) | 0 (0 to 0) |  | 376 (124 to 671) | 1.04 (0.34 to 1.86) | 0 (0 to 0) | 0 (0 to 0) |
| South Sudan | 1002 (309 to 1755) | 18.2 (5.45 to 32.96) | 1519 (450 to 2516) | 17.04 (5 to 29.17) |  | 197 (46 to 376) | 3.55 (0.87 to 6.84) | 302 (75 to 572) | 3.37 (0.86 to 6.38) |
| Spain | 0 (0 to 0) | 0 (0 to 0) | 0 (0 to 0) | 0 (0 to 0) |  | 0 (0 to 0) | 0 (0 to 0) | 0 (0 to 0) | 0 (0 to 0) |
| Sri Lanka | 7321 (3875 to 10869) | 43.64 (23.27 to 64.92) | 0 (0 to 0) | 0 (0 to 0) |  | 1414 (607 to 2377) | 8.37 (3.62 to 13.94) | 0 (0 to 0) | 0 (0 to 0) |
| Sudan | 30570 (23145 to 36847) | 168.09 (125.55 to 201.33) | 34657 (17915 to 47658) | 89.13 (48.93 to 121.07) |  | 5312 (3195 to 7951) | 29.16 (17.93 to 43.05) | 6181 (3130 to 10133) | 15.85 (8.18 to 25.58) |
| Suriname | 51 (15 to 78) | 13.55 (4.12 to 20.92) | 42 (13 to 66) | 7.08 (2.07 to 11.05) |  | 10 (2 to 18) | 2.69 (0.69 to 4.65) | 8 (2 to 16) | 1.42 (0.34 to 2.59) |
| Sweden | 0 (0 to 0) | 0 (0 to 0) | 0 (0 to 0) | 0 (0 to 0) |  | 0 (0 to 0) | 0 (0 to 0) | 0 (0 to 0) | 0 (0 to 0) |
| Switzerland | 0 (0 to 0) | 0 (0 to 0) | 0 (0 to 0) | 0 (0 to 0) |  | 0 (0 to 0) | 0 (0 to 0) | 0 (0 to 0) | 0 (0 to 0) |
| Syrian Arab Republic | 10101 (5117 to 14872) | 90.92 (48.32 to 131.08) | 9168 (4842 to 13947) | 62.04 (33.41 to 93.63) |  | 1831 (829 to 3055) | 16.4 (7.73 to 27.16) | 1699 (778 to 2861) | 11.45 (5.38 to 19.32) |
| Taiwan (Province of China) | 0 (0 to 0) | 0 (0 to 0) | 0 (0 to 0) | 0 (0 to 0) |  | 0 (0 to 0) | 0 (0 to 0) | 0 (0 to 0) | 0 (0 to 0) |
| Tajikistan | 1163 (569 to 1951) | 23.99 (11.88 to 39.5) | 1845 (971 to 2668) | 20.18 (10.76 to 28.76) |  | 223 (89 to 411) | 4.55 (1.86 to 8.23) | 365 (158 to 631) | 3.95 (1.73 to 6.72) |
| Thailand | 23910 (13804 to 34347) | 42.9 (24.95 to 61.31) | 11769 (6641 to 16828) | 14.29 (8.3 to 20.37) |  | 4414 (2162 to 7294) | 7.87 (3.95 to 12.94) | 2315 (1127 to 3929) | 2.82 (1.39 to 4.76) |
| Timor-Leste | 379 (219 to 505) | 57.96 (35.06 to 75.53) | 154 (93 to 220) | 13.76 (8.36 to 19.45) |  | 67 (36 to 104) | 10.18 (5.58 to 15.83) | 30 (13 to 52) | 2.68 (1.22 to 4.56) |
| Togo | 1572 (665 to 2703) | 45.98 (19.06 to 79.44) | 2536 (1173 to 3911) | 33.74 (15.18 to 52.37) |  | 300 (106 to 556) | 8.71 (2.98 to 16.24) | 501 (194 to 867) | 6.63 (2.56 to 11.66) |
| Tokelau | 0 (0 to 0) | 5.11 (3.33 to 7.52) | 0 (0 to 0) | 0 (0 to 0) |  | 0 (0 to 0) | 0.96 (0.5 to 1.59) | 0 (0 to 0) | 0 (0 to 0) |
| Tonga | 3 (2 to 5) | 4.26 (2.73 to 6.02) | 0 (0 to 0) | 0 (0 to 0) |  | 1 (0 to 1) | 0.82 (0.43 to 1.35) | 0 (0 to 0) | 0 (0 to 0) |
| Trinidad and Tobago | 168 (30 to 304) | 14.2 (2.63 to 25.58) | 104 (13 to 199) | 6.86 (0.74 to 13.38) |  | 33 (5 to 66) | 2.79 (0.45 to 5.5) | 21 (2 to 44) | 1.37 (0.12 to 2.95) |
| Tunisia | 2595 (1203 to 3958) | 33.49 (16.54 to 49.49) | 1427 (878 to 2006) | 11.78 (7.31 to 16.64) |  | 465 (184 to 822) | 5.97 (2.48 to 10.38) | 277 (135 to 471) | 2.28 (1.12 to 3.87) |
| Turkey | 35275 (17030 to 58455) | 61.56 (30.53 to 101.03) | 26492 (12842 to 37977) | 30.5 (14.88 to 43.52) |  | 6841 (2953 to 12173) | 11.83 (5.13 to 21.02) | 5257 (2205 to 8947) | 6.05 (2.53 to 10.31) |
| Turkmenistan | 0 (0 to 0) | 0 (0 to 0) | 0 (0 to 0) | 0 (0 to 0) |  | 0 (0 to 0) | 0 (0 to 0) | 0 (0 to 0) | 0 (0 to 0) |
| Tuvalu | 1 (1 to 1) | 11.57 (7.01 to 16.01) | 0 (0 to 0) | 3.02 (1.98 to 4.32) |  | 0 (0 to 0) | 2.03 (1.14 to 3.25) | 0 (0 to 0) | 0.57 (0.3 to 0.95) |
| Uganda | 10071 (5915 to 14080) | 70.12 (40.46 to 99.38) | 8550 (4463 to 12364) | 24.95 (12.19 to 36.69) |  | 1794 (860 to 2910) | 12.49 (6.09 to 20.59) | 1694 (737 to 2838) | 4.92 (2.03 to 8.24) |
| Ukraine | 8644 (4315 to 12558) | 15.99 (7.77 to 23.27) | 8360 (4165 to 11931) | 18.34 (9.09 to 26.26) |  | 1696 (672 to 2861) | 3.14 (1.19 to 5.33) | 1636 (698 to 2743) | 3.61 (1.46 to 6.12) |
| United Arab Emirates | 0 (0 to 0) | 0 (0 to 0) | 0 (0 to 0) | 0 (0 to 0) |  | 0 (0 to 0) | 0 (0 to 0) | 0 (0 to 0) | 0 (0 to 0) |
| United Kingdom | 0 (0 to 0) | 0 (0 to 0) | 0 (0 to 0) | 0 (0 to 0) |  | 0 (0 to 0) | 0 (0 to 0) | 0 (0 to 0) | 0 (0 to 0) |
| United Republic of Tanzania | 7747 (4010 to 11197) | 37.26 (19.84 to 53.09) | 6872 (3787 to 9906) | 14.67 (7.79 to 21.15) |  | 1373 (627 to 2341) | 6.6 (3.18 to 11.14) | 1363 (569 to 2317) | 2.9 (1.2 to 4.96) |
| United States of America | 0 (0 to 0) | 0 (0 to 0) | 0 (0 to 0) | 0 (0 to 0) |  | 0 (0 to 0) | 0 (0 to 0) | 0 (0 to 0) | 0 (0 to 0) |
| United States Virgin Islands | 8 (2 to 13) | 7.64 (1.65 to 12.32) | 3 (1 to 6) | 2.99 (0.3 to 5.46) |  | 2 (0 to 3) | 1.52 (0.25 to 2.75) | 1 (0 to 1) | 0.6 (0.05 to 1.22) |
| Uruguay | 0 (0 to 0) | 0 (0 to 0) | 0 (0 to 0) | 0 (0 to 0) |  | 0 (0 to 0) | 0 (0 to 0) | 0 (0 to 0) | 0 (0 to 0) |
| Uzbekistan | 5583 (2663 to 9447) | 28.78 (14.03 to 47.93) | 5253 (2528 to 7873) | 15.78 (7.88 to 23.48) |  | 1066 (428 to 1949) | 5.45 (2.25 to 9.84) | 1034 (410 to 1767) | 3.08 (1.24 to 5.26) |
| Vanuatu | 35 (20 to 54) | 30.15 (17.14 to 45.42) | 54 (33 to 80) | 21.45 (13.25 to 31.4) |  | 7 (3 to 11) | 5.59 (2.63 to 9.17) | 10 (5 to 17) | 4.06 (2 to 6.6) |
| Venezuela (Bolivarian Republic of) | 2070 (398 to 3390) | 11.67 (2.6 to 18.84) | 3643 (1048 to 5882) | 12.58 (3.52 to 20.33) |  | 413 (63 to 779) | 2.33 (0.5 to 4.27) | 724 (181 to 1336) | 2.5 (0.61 to 4.64) |
| Viet Nam | 37511 (22042 to 51090) | 60.88 (36.11 to 83) | 20164 (9324 to 29287) | 19 (8.9 to 27.49) |  | 6834 (3563 to 10971) | 11.05 (5.86 to 17.6) | 3998 (1489 to 6834) | 3.76 (1.42 to 6.45) |
| Yemen | 17402 (10359 to 22762) | 146.83 (91.49 to 191.97) | 36276 (18881 to 53049) | 121.68 (65.61 to 174.69) |  | 3074 (1658 to 4852) | 25.85 (14.23 to 40.36) | 6573 (3057 to 10865) | 21.9 (10.71 to 35.69) |
| Zambia | 2830 (1249 to 4639) | 41.02 (18.34 to 67.77) | 3690 (1618 to 5545) | 21.83 (9.1 to 33.92) |  | 524 (203 to 956) | 7.52 (2.95 to 13.48) | 727 (257 to 1243) | 4.29 (1.47 to 7.58) |
| Zimbabwe | 0 (0 to 0) | 0 (0 to 0) | 0 (0 to 0) | 0 (0 to 0) |  | 0 (0 to 0) | 0 (0 to 0) | 0 (0 to 0) | 0 (0 to 0) |
