# Supplementary figures and images for: The global burden, trends, and inequalities of individuals with developmental and intellectual disabilities attributable to iodine deficiency from 1990 to 2019 and its prediction up to 2030
Source: Front Nutr. 2024 Jun 17;11:1366525. doi: 10.3389/fnut.2024.1366525 (PMC11215058; doi:10.3389/fnut.2024.1366525)

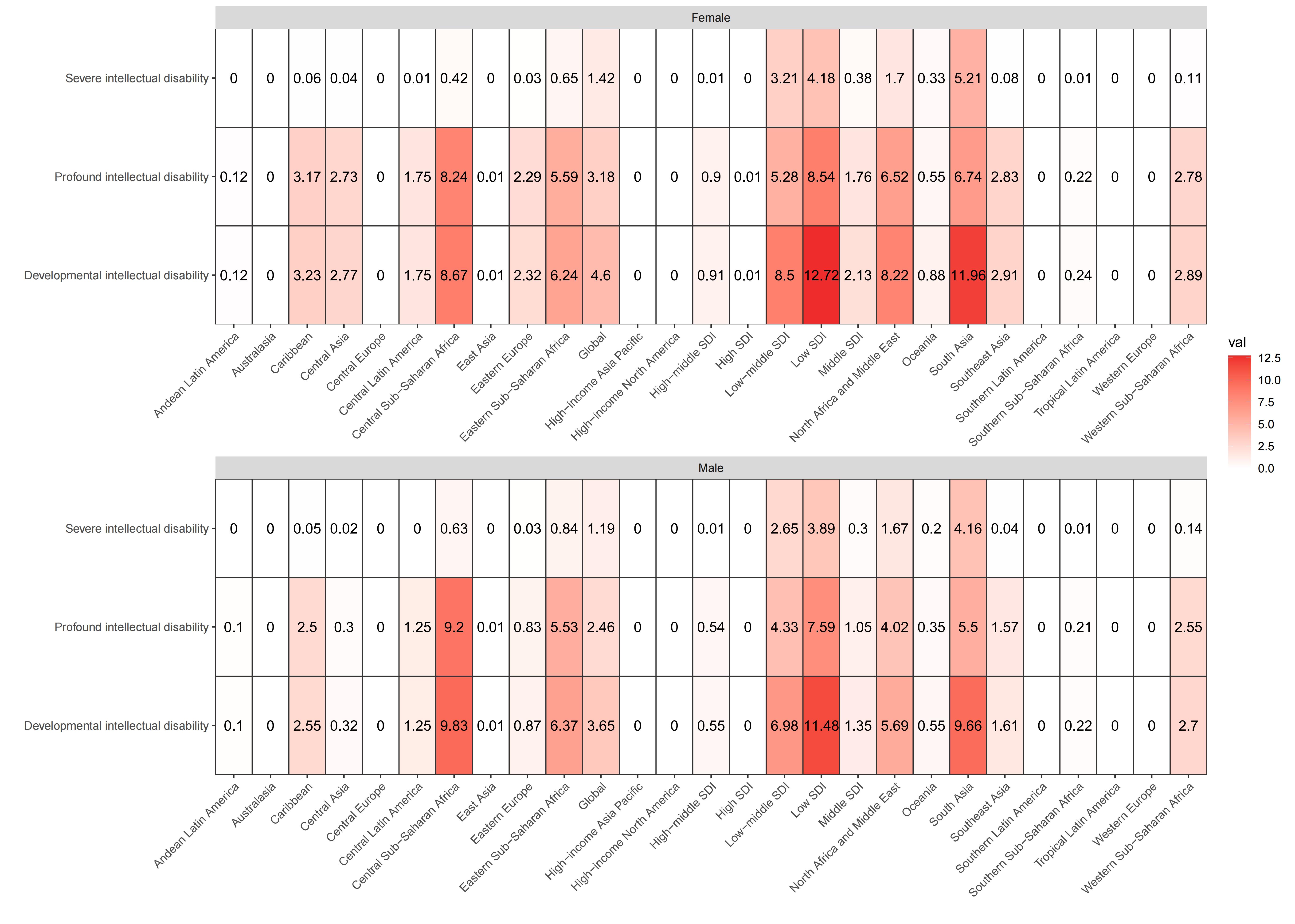

Supplement: Supplementary Figure 1 — These heatmap show the ASYR of developmental intellectual disability attributable to iodine deficiency in GBD regions by sex and severity categories in 2019. The shade of color of each block in the heatmap represents the size of the numerical value, and the figure inside represents the absolute number of the age-standardized prevalence. ASYR, age standardized YLDs rate. [file Image_1.JPEG]

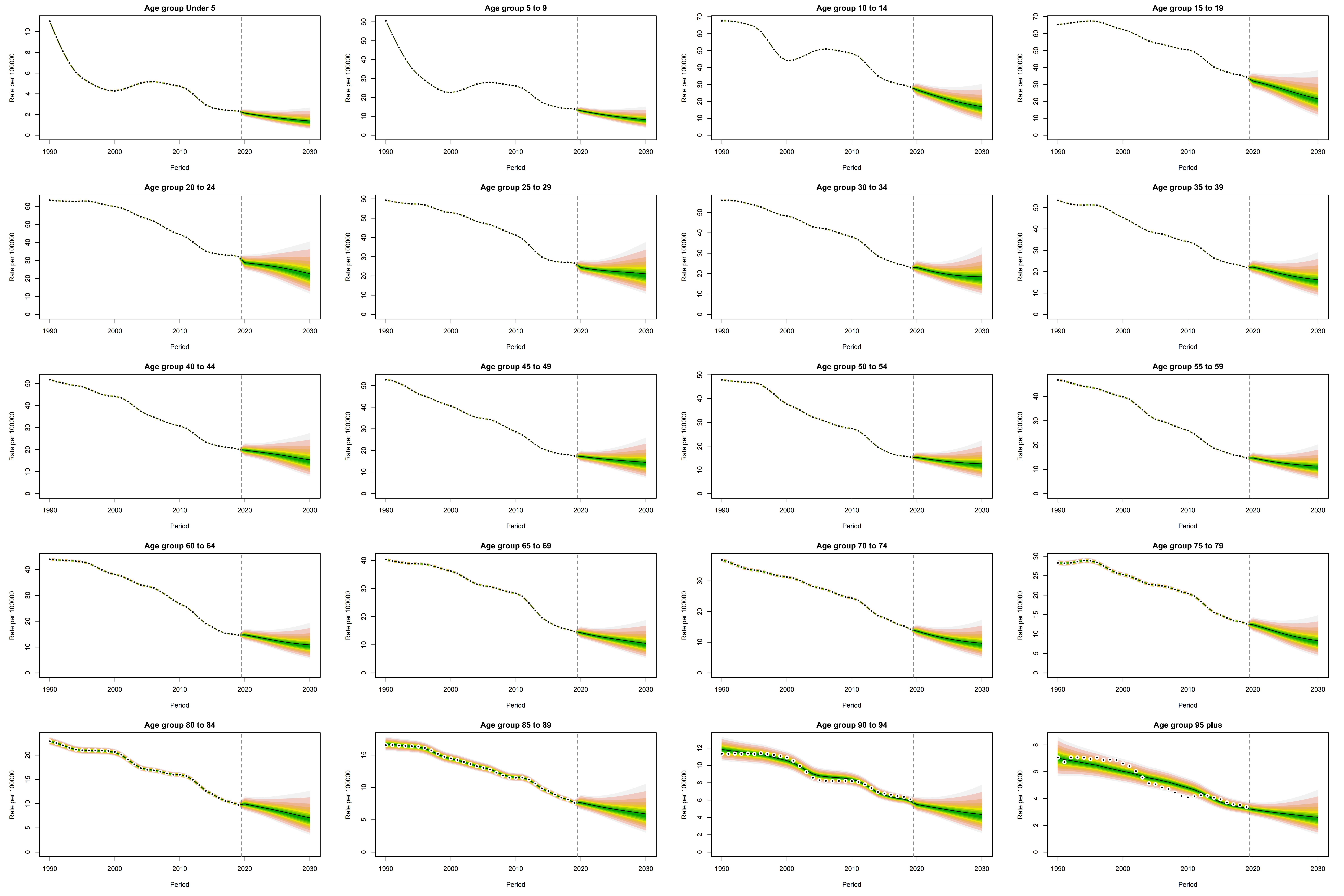

Supplement: Supplementary Figure 2 — The projections of ASPR by age for males of developmental intellectual disability attributable to iodine deficiency from 2020 to 2030. ASPR, age-standardized prevalence rate. [file Image_2.JPEG]

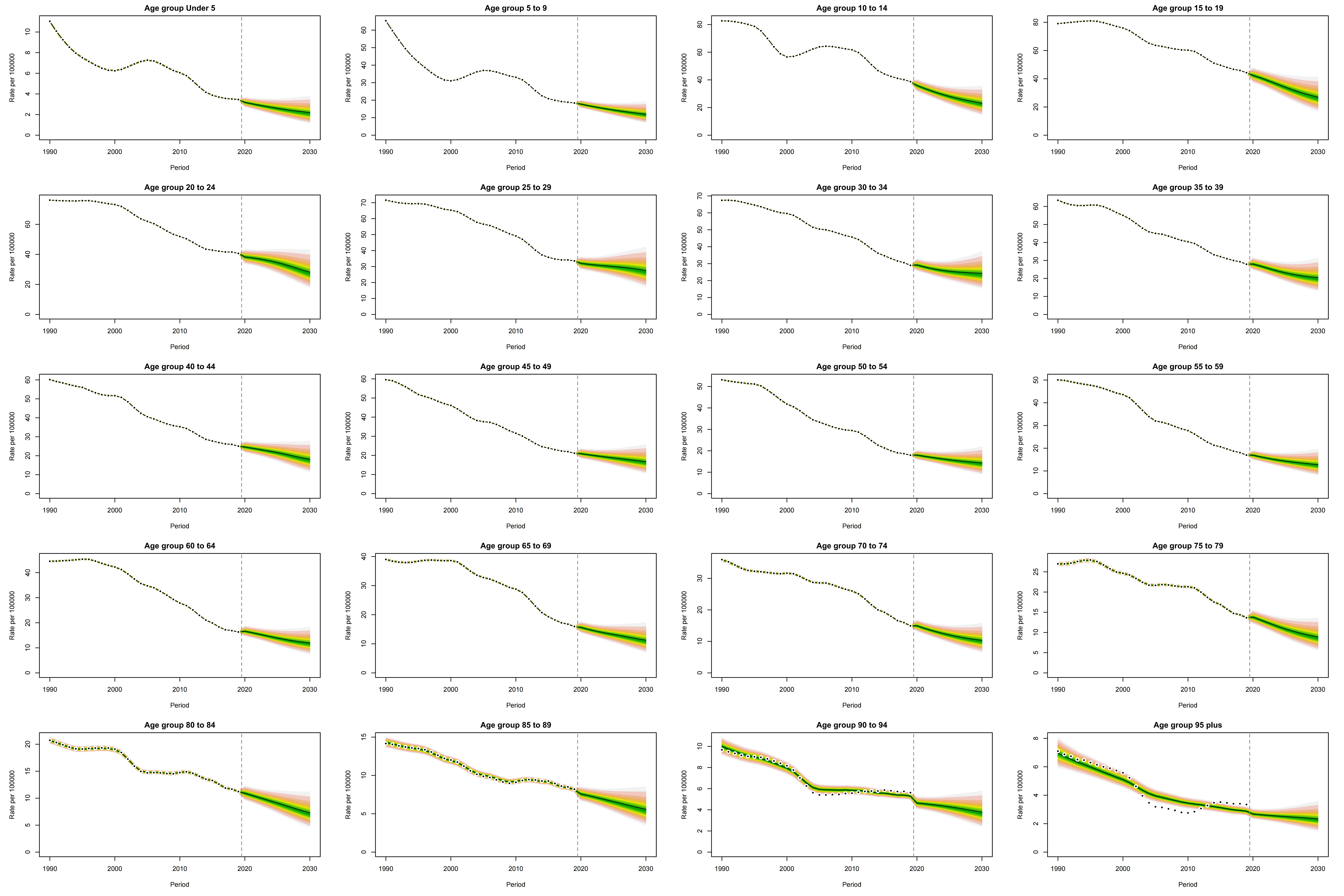

Supplement: Supplementary Figure 3 — The projections of ASPR by age for females of developmental intellectual disability attributable to iodine deficiency from 2020 to 2030. ASPR, age-standardized prevalence rate. [file Image_3.JPEG]

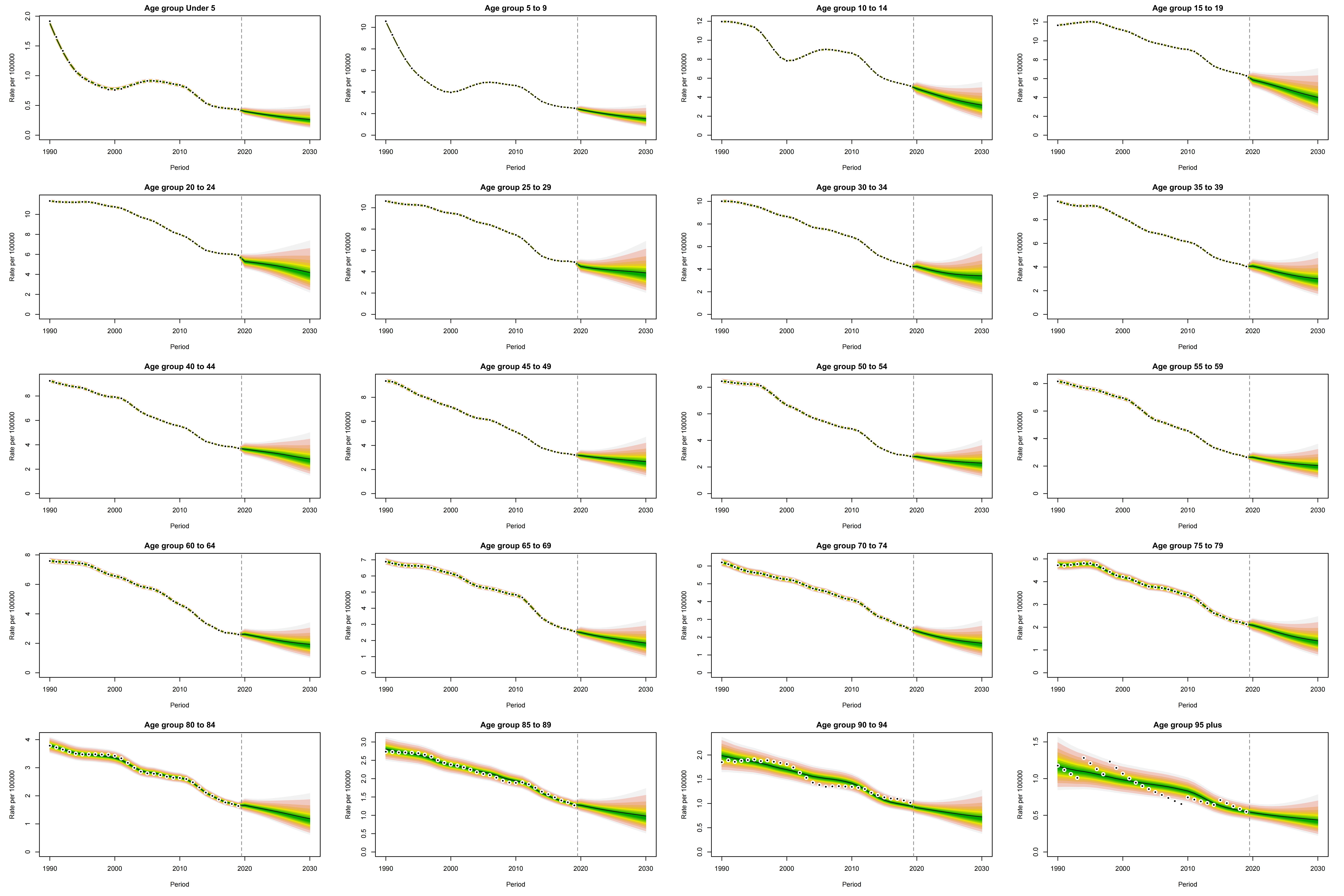

Supplement: Supplementary Figure 4 — The projections of ASYR by age for males of developmental intellectual disability attributable to iodine deficiency from 2020 to 2030. ASYR, age standardized YLDs rate. [file Image_4.JPEG]

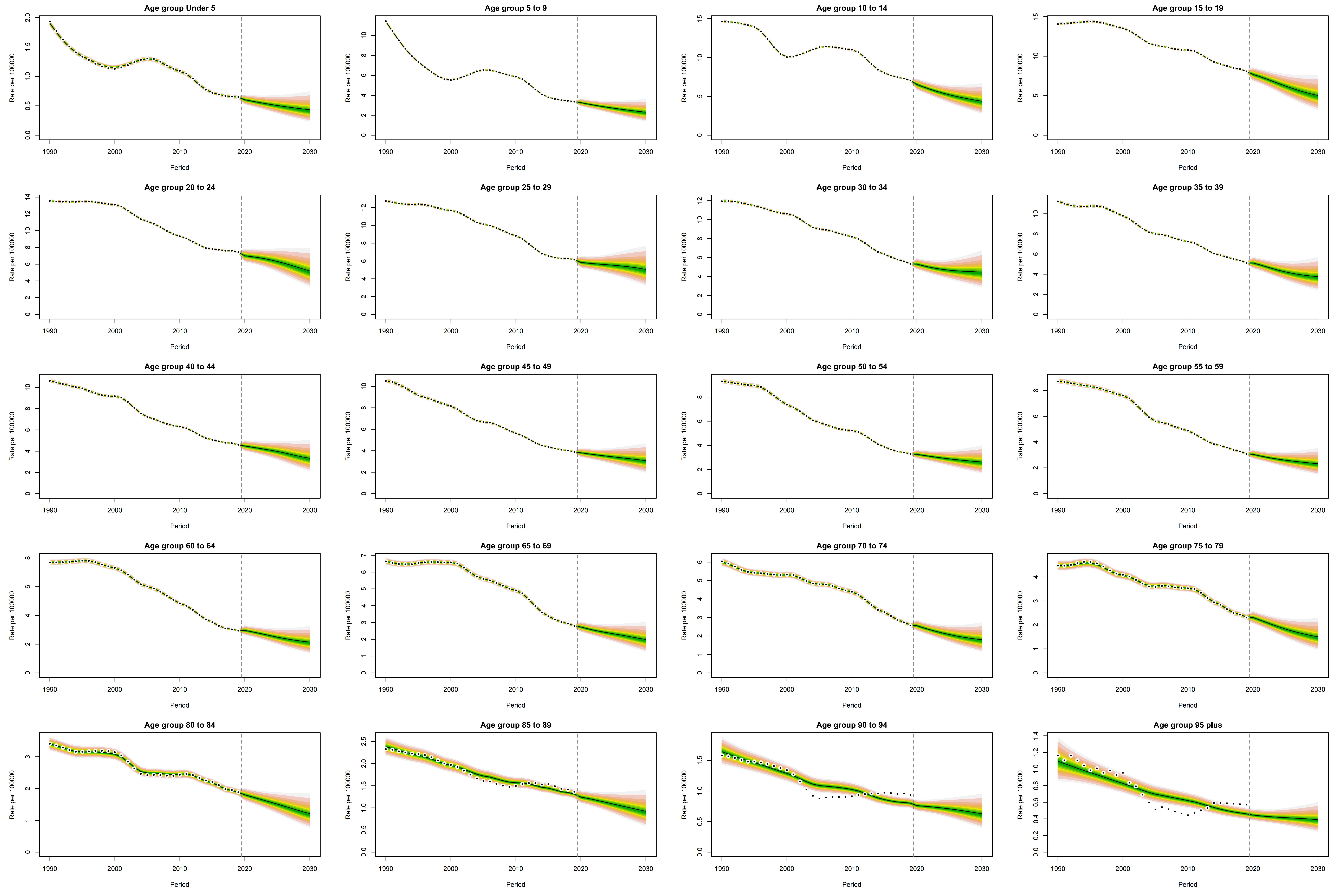

Supplement: Supplementary Figure 5 — The projections of ASYR by age for females of developmental intellectual disability attributable to iodine deficiency from 2020 to 2030. ASYR, age standardized YLDs rate. [file Image_5.JPEG]
